# Solvent-Mediated, Reversible Ternary Graphite Intercalation Compounds for Extreme-Condition Li-Ion Batteries

**DOI:** 10.1021/jacs.4c04594

**Published:** 2024-06-07

**Authors:** Lei Tao, Dawei Xia, Poom Sittisomwong, Hanrui Zhang, Jianwei Lai, Sooyeon Hwang, Tianyi Li, Bingyuan Ma, Anyang Hu, Jungki Min, Dong Hou, Sameep Rajubhai Shah, Kejie Zhao, Guang Yang, Hua Zhou, Luxi Li, Peng Bai, Feifei Shi, Feng Lin

**Affiliations:** † Department of Chemistry, Virginia Tech, Blacksburg, Virginia 24061, United States; ‡ Department of Energy, Environment & Chemical Engineering, Washington University in St. Louis, St. Louis, USA, Missouri 63130, United States; § Department of Energy and Mineral Engineering, The Pennsylvania State University, University Park, Pennsylvania 16802, United States; ∥ Center for Functional Nanomaterials, Brookhaven National Laboratory, Upton, New York 11973, United States; ⊥ X-Ray Science Division, Argonne National Laboratory, Lemont, Illinois 60439, United States; # Mechanical Engineering, Purdue University, West Lafayette, Indiana 47907, United States; ∇ Chemical Sciences Division, Oak Ridge National Laboratory, Oak Ridge, Tennessee 37830, United States; ○ Department of Materials Science and Engineering, Virginia Tech, Blacksburg, Virginia 24061, United States

## Abstract

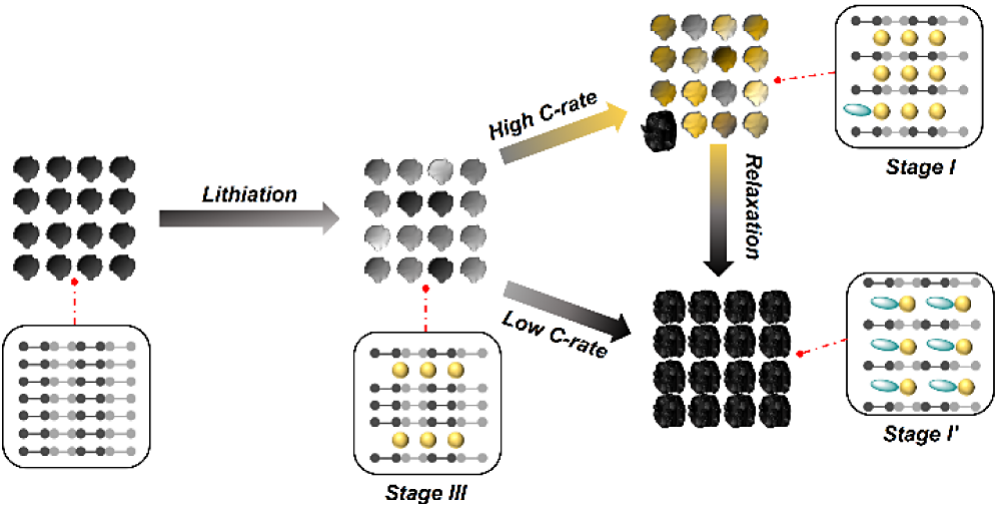

Traditional Li-ion intercalation chemistry into graphite anodes exclusively utilizes the cointercalation-free or cointercalation mechanism. The latter mechanism is based on ternary graphite intercalation compounds (t-GICs), where glyme solvents were explored and proved to deliver unsatisfactory cyclability in LIBs. Herein, we report a novel intercalation mechanism, that is, in situ synthesis of t-GIC in the tetrahydrofuran (THF) electrolyte via a spontaneous, controllable reaction between binary-GIC (b-GIC) and free THF molecules during initial graphite lithiation. The spontaneous transformation from b-GIC to t-GIC, which is different from conventional cointercalation chemistry, is characterized and quantified via operando synchrotron X-ray and electrochemical analyses. The resulting t-GIC chemistry obviates the necessity for complete Li-ion desolvation, facilitating rapid kinetics and synchronous charge/discharge of graphite particles, even under high current densities. Consequently, the graphite anode demonstrates unprecedented fast charging (1 min), dendrite-free low-temperature performance, and ultralong lifetimes exceeding 10 000 cycles. Full cells coupled with a layered cathode display remarkable cycling stability upon a 15 min charging and excellent rate capability even at −40 °C. Furthermore, our chemical strategies are shown to extend beyond Li-ion batteries to encompass Na-ion and K-ion batteries, underscoring their broad applicability. Our work contributes to the advancement of graphite intercalation chemistry and presents a low-cost, adaptable approach for achieving fast-charging and low-temperature batteries.

## Introduction

Graphite intercalation compounds (GICs) have shown programmable physiochemical properties for applications such as electrical conductors, catalysis, hydrogen storage, and energy storage.^1−3^ Among them, alkali-ion based GICs are particularly attractive as electrodes for rechargeable batteries.^1,4^ GICs exhibit tunable guest–host interactions with anions, cations, and solvated cations, enabling different battery chemistries.^5,6^ One of the most extensively studied GICs is lithiated graphite (LiC_6_), a binary GIC (b-GIC) serving as the anode in commercial Li-ion batteries (LIBs).^7^ Researchers synthesized LiC_6_ from graphite via chemical means in the 1950s,^8^ but it took over three decades to synthesize it electrochemically.^9−11^ The electrochemical synthesis is more intricate, governed by the solvation structure, the thermodynamic properties of electrolytes, and the graphite–electrolyte electrochemical interphase.^12^ In conventional graphite intercalation chemistry, the interphase allows cations to transport while blocking other electrolyte components such as solvent molecules.^13^ Ternary-GICs (t-GICs) are another category of GICs, which have cations and solvent molecules cointercalated and offer excellent chemical and structural tunability.^14^ Typically, a single reaction can only lead to either b-GIC or t-GIC, and the coexistence of and interconversion between b-GIC and t-GIC have not been reported in battery chemistry.

Bidentate ligands such as dimethoxyethane (DME) have strong chelating effects with Li ions, resulting in facile Li-solvent cointercalation upon electrochemical cycling and chemical prelithiation of graphite.^15,16^ Unidentate cyclic ether ligands such as tetrahydrofuran (THF) have unique electrochemical and chemical interactions with graphite, which can potentially enable dynamic interconversion between b-GICs and t-GICs even under battery operating conditions. When solvating Li ions, THF has a higher coordination number than bidentate ligands,^17^ suggesting lower desolvation energy and less tendency to be cointercalated with cations during electrochemical reactions. However, the earlier literature reported the ternarisation of stage 1 KC_24_ in the THF solvent leading to the formation of a stage 2 K(THF)_2_C_20–30_ t-GIC (i.e., chemical reaction).^18^ Therefore, such a discrepancy between electrochemical and chemical reactions implies that hybrid intercalation and cointercalation reactions are possible, which can then offer a dynamic interconversion between b-GICs and t-GICs.

The dynamic interconversion between b-GICs and t-GICs is far beyond advancing the fundamental insights into intercalant-graphite interactions and shows great practical promise for designing extreme-condition batteries.^19^ t-GICs have outstanding electrical conductivity and fast diffusion of solvated ions between graphene layers.^14^ Furthermore, the energy barrier of the charge transfer process is lowered due to the cointercalation mechanism.^6,20,21^ In fact, a plethora of Li-t-GICs have been explored by using glyme solvents to make use of these advantages, but the cycling stability issues have remained unresolved, which can be due to the large volume expansion of lithiated graphite (>200%).^15^ Given the weaker interaction with Li-ion compared to glyme solvents, we hypothesize that unidentate ligand THF can be an alternative to ignite new cointercalation chemistry for extreme-condition batteries, especially for fast-charge and low-temperature applications. Last but not least, THF brings tremendous cost reduction as an electrolyte solvent compared to commercial electrolyte solvents and other solvents under development.

Herein, we report a t-THF-GIC displaying an ultralong lifespan of over 10 000 cycles with high Coulombic efficiency (CE) and negligible capacity decay, one min fast charging (100% retention), and unprecedented low-temperature performance without Li plating. Such new cointercalation chemistry with high stability has never been achieved in traditional glyme solvents. We discovered in situ b-GIC to t-GIC transformation during the initial cycles, which can be modulated by current density, temperature, SEI properties, and graphite type. The in situ chemical synthesis of t-THF-GICs can proceed through uptaking THF molecules from the electrolyte in the absence of a dense and continuous SEI. Paired with the NMC cathode, the full cell displays outstanding cycling stability with a 15 min charging. Even at −40 °C, the excellent rate capability can be achieved. The graphite anode with such an innovative operation mechanism has led to the best performance cointercalation based batteries reported thus far. Our study has enriched the knowledge library for graphite intercalation chemistry through a suite of operando characterization and represents a leap for Li-ion chemistry operating under extreme conditions.

## Results and Discussion

### Chemical Reaction between B-GICs and THF Hidden during the First Cycle

**Figure 1:**
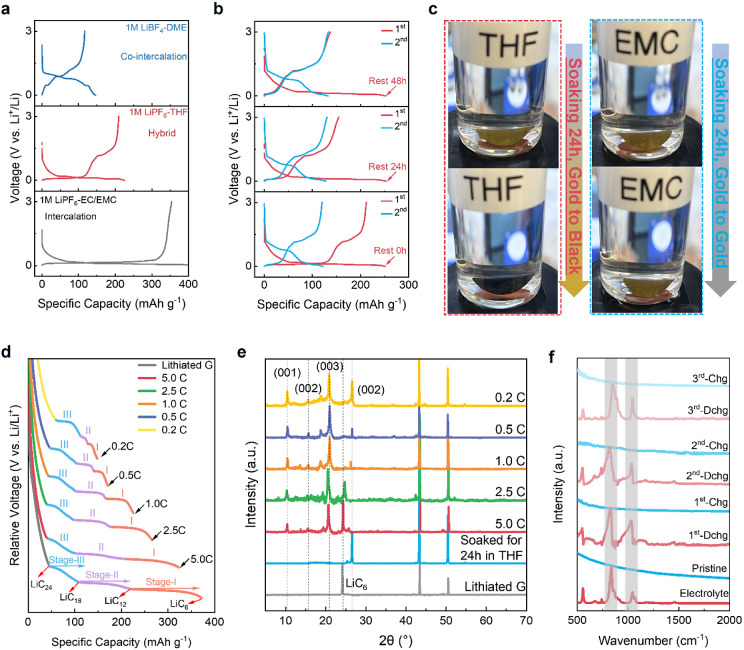
Chemical reaction between b-GICs and THF hidden during the first cycle in THF-based electrolytes. a) Discharge–charge curves of the graphite anode cycled in 1 M LiPF_6_-EC/EMC (cointercalation-free), 1 M LiPF_6_-THF (cointercalation-free to cointercalation), and 1 M LiBF_4_-DME (cointercalation) at 1C (1C = 100 mA g^–1^). b) Discharge–charge curves of the graphite anode cycled in the 1 M LiPF_6_-THF electrolyte with different resting durations after the 1st lithiation (0, 24, and 48 h). c) Photographs of fully lithiated graphite (LiC_6_) before and after soaking in THF and EMC solvents. d) The 1st discharge curves of the graphite anode cycled in the 1 M LiPF_6_-THF electrolyte at a current range of 0.2 to 5 C. A graphite with a complete lithiation process, showing typical three evolution stages (III, II, and I), is provided as a comparison to observe the differences in the three stages under different currents. e) XRD patterns of the graphite anodes after the 1st discharge shown in c and d. f) FTIR spectroscopy of the graphite anodes cycled in the 1 M LiPF_6_-THF electrolyte and measured after different cycling histories (Notably, after the 1st discharge, the cell was rested for 1 day before being dissembled).

We use superior graphite as the platform, and Figure S1 shows the morphology and structure details. A graphite anode has been perceived to reversibly store mobile ions in a single storage mechanism, that is, either intercalation or cointercalation. Figure 1a demonstrates the intercalation in the EC/EMC-based electrolyte and the cointercalation in the DME-based electrolyte. The voltage profiles and the corresponding d*Q*/d*V* curves show common intercalation or cointercalation plateaus (Figure 1a and Figure S1). When using a 1 M LiPF_6_-THF electrolyte, we observe interesting phenomena: (1) the first lithiation shows a cointercalation-free mode, while the first delithiation is a combination of cointercalation and intercalation (Figure 1a). The corresponding d*Q*/d*V* curves are asymmetrical (Figure S2). (2) The second lithiation is predominantly cointercalation and is highly reversible (Figure 1b). The cointercalation-free feature can be explained by the weak Li^+^-THF binding (discussed in MD simulation), especially compared to glyme counterparts. The cointercalation contribution during the first delithiation can only arise from the chemical reaction between b-GIC and THF molecules in the electrolyte. The underlying b-GIC to t-GIC transformation will be discussed in a subsequent section. In this section, we present electrochemical analyses to elucidate the in situ formation of t-GICs in operating batteries. We first demonstrate the tailorable Li storage mechanism in the first cycle. As shown in Figure 1b, the capacity contribution from deintercalating Li ions decreases if we rest the cell after the first lithiation. Especially after a 48 h rest, the capacity contribution from deintercalating Li ions disappears, and the charging curve overlaps with the second charge. These results show that the in situ b-GIC to t-GIC transformation is not completed immediately following the formation of b-GIC.

Then, we investigate how in situ transformation is governed by the reaction environment. We first study the pure chemical reaction without the influence of the electrical field by soaking lithiated graphite (i.e., LiC_6_) in THF and EMC solvents. We observe a golden-to-black color change only in the THF solvent after 24 h (Figure 1c). In battery cells, the discharge capacity anomalously increases as the current density increases from 0.2 to 5 C (Figure 1d and Figure S2c). Specifically, the stage II to stage I conversion of lithiated graphite is less pronounced when a low current density is used (Figure 1d and Figure S1b,c). When the cells are rested after the initial discharge, a decrease in the following charge capacity is consistently observed, especially for the cells cycled at high current densities (Figure S 2d). We conjecture that at low current densities or during the rest, there is ample time for THF to react with b-GICs. Once b-GICs transform into t-GICs, they cannot proceed to form b-GICs in a lower stage. Considering the diverse morphologies of graphite materials, we also investigated the b-GIC to t-GIC transformation using mesocarbon microbeads (MCMBs) and natural graphite (NG), as shown in Figure S3. We find that the cycle numbers needed for the transformation is different. MCMB requires more cycles to reach a stable capacity (complete transition of b-GIC to t-GIC). Another finding is that the ultimate capacity is identical in different graphite materials (Figure S3c). These data support our hypothesis that b-GIC reacts with free THF to form t-GIC.

The XRD patterns further show that the LiC_6_ characteristic peaks weaken after soaking in THF or cycling at low current densities (Figure 1e). We conclude that the b-GIC can have two phase transformation pathways: (1) further electrochemical lithiation to form lower-stage b-GIC and (2) chemical reaction with free THF molecules in the electrolyte. At a low current density, the reactions are more concurrent, leading to more formation of t-GICs. Interestingly, the same chemical reaction also occurs in the G||Na system (Figure S4) and even in the G||K system (Figure S5), breaking the conventional wisdom that cyclic ether cannot reversibly cointercalate into graphite.^22,23^

The in situ formation of t-GIC can be affected by the SEI composition and thickness, which are associated with the stability of salts and solvents. When we switch from LiPF_6_ to LiFSI, more LiC_6_ is formed during the first lithiation, but the b-GIC to t-GIC transformation is less pronounced (Figure S6a-b). The cointercalation behavior still occurs, but irreversibly, leading to rapid capacity decay of the G||Li cell (Figure S6c). We observe thicker SEIs cycled in the 1 M LiFSI-THF electrolyte, which hampers the interaction between b-GIC and free THF molecules (Figure S7). The graphite anode cycled in 1 M LiPF_6_-THF exhibits clean surfaces without SEI formation or ultrathin SEI sporadically distributed, consistent with its high ICE (Figure S2c, S8a-e). The clean graphite surface explains the high reversibility of the Li^+^-THF cointercalation. Such differences in SEI properties led to drastically different kinetics. The desolvation energy and the energy for Li^+^ transport through SEI are shown in Figure S9. The desolvation energies of the two electrolytes remain at almost the same value, i.e., 34 vs 32 kJ mol^–1^ (Figure S9c). This is also consistent with the voltage profile of the G||Li cell that incomplete desolvation leads to solvent cointercalation into graphite. However, the activation energy for Li^+^ transport through SEIs in the LiPF_6_-THF electrolyte is significantly lower than that in the LiFSI-THF electrolyte (Figure S9d).

We then examine the structural changes of graphite due to the b-GICs to t-GICs transformation using ex situ FTIR and Raman spectroscopy. Compared with pristine graphite, the Li^+^-THF characteristic FTIR peaks emerge after full discharge (including the first discharge), and then disappear after full charge (Figure 1f). This directly manifests the uptake of free THF molecules and the b-GIC to t-GIC transformation. Meanwhile, Li^+^-THF cointercalation is highly reversible. Figure S10 shows the ex situ Raman spectroscopy of graphite during cycling. The pristine graphite exhibits a weak D band (1350 cm^–1^) and a strong G band (1600 cm^–1^), with an *I*_D_/*I*_G_ ratio of 0.25. During the first discharge, the intensity of the D band increases significantly, implying that the well-ordered graphitic interlayers become disordered due to structural changes caused by THF uptake. During the first charge, the intensity of the D and G bands cannot return to their original state. The same phenomenon is observed in the second and third cycles, while the *I*_D_/*I*_G_ ratio increases from 0.25 to 0.28, suggesting an increase in structural disorder attributable to repeated Li^+^-THF cointercalation. Operando optical microscopy of graphite during cycling is shown in Video S1. Only a slight yellowish change occurs during the first discharge, indicating the presence of LiC_6_, while in subsequent cycles, the graphite remains black with volume expansion, verifying the cointercalation behavior (Video S1).

### Operando Characterization of the B-GIC to T-GIC Transformation Mechanism

**Figure 2:**
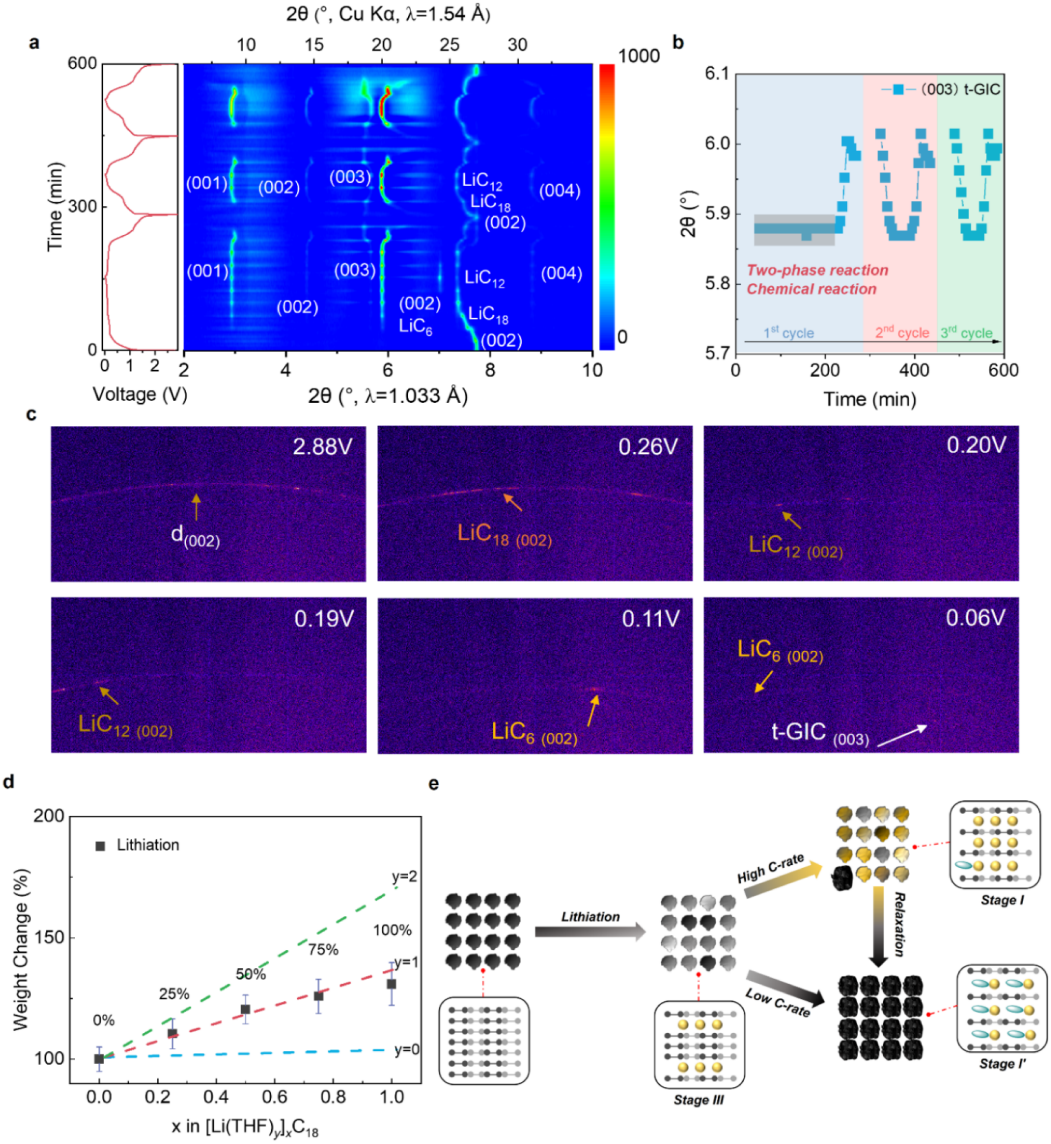
Operando characterization of the b-GIC to t-GIC transformation mechanism. a), Operando synchrotron XRD patterns and galvanostatic charge–discharge curves of the G||Li cell at 1C for 3 cycles. b) The evolution of the t-GIC (003) peak during the first 3 cycles. c) CMCD to show the synchronous discharging (1st discharge) among different graphite particles. d) Measurements of the weight changes of the graphite at different discharge states to estimate the number of cointercalated THF. The error bars are created based on the standard deviation of three repeated measurements. e) Schematic illustration of the in situ t-GIC synthesis during the 1st lithiation.

The structural evolution of graphite is further investigated by operando synchrotron XRD (Figure 2a, Figure S11). The pristine graphite displays a strong (002) diffraction peak at 7.7°. During the first discharge, graphite undergoes lithiation to form b-GIC LiC_18_ and LiC_12_, as evidenced by the shift of the (002) peak toward low angles. At stage II (∼0.12 V), Li^+^-THF t-GIC gradually emerges, as shown by three new peaks at lower angles and one new peak at higher angles, corresponding to the (001), (002), (003), and (004) planes of graphite, respectively. Since the lithiation voltage profile shows no cointercalation feature between 0.4 and 0.7 V, the emergence of Li^+^-THF t-GIC is caused by the chemical reaction between b-GIC and free THF molecules. Upon the subsequent charge, these new peaks shift back reversibly and disappear at the end of the charge. However, the (002) peak is weakened due to the increasing structural disorder. Such a reversible evolution of the (002) peak is observed in the subsequent cycles, but the LiC_6_ peak no longer appears (Figure S11), suggesting a pure Li^+^-THF cointercalation behavior with excellent reversibility. Figure 2b delineates the position of the t-GIC (003) peak along with the cycling time. The t-GIC (003) peak at 5.87° emerges at 52.7 min during discharging due to the b-GIC to t-GIC transformation. The asymmetric behavior of structural evolution in the first cycle agrees well with the asymmetric voltage profile and the proposed in situ b-GIC to t-GIC transformation. After the second and third cycles, the peak shift is highly symmetric, consistent with excellent reversibility of Li^+^-THF cointercalation. The ex-situ XRD patterns further characterize the evolution of graphite diffraction peak after the 10th and 100th cycles, where the (002) peak of both shows similar intensities (Figure S12). These results demonstrate that the Li^+^-THF cointercalation in the graphite is highly reversible, and the interlayer structure of graphite is highly stable during extended cycling.

This transformation mechanism completely differs from the traditional electrochemical pathway to form t-GICs (i.e., DME or diglyme cointercalation).^15,24^ It is well-known that DME has a strong chelating effect with a Li ion. As such, a t-GIC directly forms during the first lithiation process (Figure 1a, Figure S13).^24^ The lithiated graphite (LiC_6_) soaking experiment also shows the difference between THF and DME in transforming b-GIC to t-GIC (Figure S14). After 1 day of soaking, the color of both LiC_6_ electrodes turned black, confirming the chemical reaction between LiC_6_ and free solvents. However, the electrode soaked in THF remained on the current collector even when shaken vigorously, while the electrode soaked in DME got peeled off and pulverized completely (Figure S14). These results indicate that the reaction between THF and b-GIC is milder, and the volume expansion of t-THF-GIC is much smaller than that of t-DME-GIC. This is also consistent with the XRD result (Figure S13). The XRD patterns of specific stages are extracted from the two operando XRD (Figure S13b). We calculated the volume expansion of t-GICs formed from these two electrolytes (Figure S13c). The t-THF-GIC has a smaller interlayer spacing Ic = 0.88 nm and a much lower volume expansion (163%) compared to the t-DME-GIC counterpart. Figure S13d compares the maximum intensity of (003), which indicates the fraction of t-GIC over b-GIC. In the THF-based electrolyte the value gradually increases with cycle number, while in the DME-based electrolyte, it directly reaches a high value because the Li^+^-DME cointercalation happens in the very first lithiation. These results show that the smaller volume expansion in t-THF-GIC and the gradual b-GIC to t-GIC transformation enable better mechanical properties for the electrode. Moreover, the Li^+^-DME cointercalation has a significantly lower diffusion coefficient in graphite than the Li^+^-THF cointercalation, resulting in a larger Li^+^-DME concentration gradient in graphite and creating a much larger mismatch strain (more discussion is shown in Figure S15).

The operando coherent X-ray multicrystal diffraction (CMCD) technique can capture the structural transformation of graphite particles with each diffraction spot representing a particle. We used CMCD to monitor if these particles have synchronous reactions (Figure 2c, Video S2). In Figure 2c, the image captured at the open circuit voltage (OCV) shows a single ring composed of sequential bright diffraction spots, which correspond to *c*-axis reflections of tens of graphite particles. During the first discharge, the bright spots shift synchronously and then gradually weaken due to the formation of b-GICs (stage III). Upon further lithiation, some b-GICs (stage III) can transform to higher-stage b-GICs (stage I, LiC_6_), whereas some b-GICs transform to t-GICs by chemically reacting with THF. Such a buffer transition path makes the volume expansion of graphite smaller, thus achieving a stabler fast-charging electrode, which also differs from the traditional DME cointercalation (Figure S16). In subsequent cycles, the weak ring moves reversibly, indicating that the cointercalation is highly reversible (Video S2). We further determine the stoichiometry of t-GIC based on the following equation:
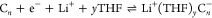


According to the voltage profile of the G||Li cell (Figure 1b), the measured reversible capacity of graphite after full discharge is ∼120 mAh g^–1^, indicating that one Li^+^(THF)_*y*_ complex contains 18 carbon atoms. We then estimate the *y* value based on the mass change of graphite at different states of discharge and determine that one THF molecule is cointercalated with one Li^+^ (Figure 2d). To provide additional support, we also added TGA experiments to quantify the number of THF in the ultimate cointercalated structure.^18^ After calculating the mass loss, we found that only one solvent was involved in t-THF-GIC after transformation (Figure S17).

In summary, we have systematically demonstrated the in situ transformation of b-GICs to t-GICs under battery operating conditions, which is now schematically described in Figure 2e. The THF electrolyte offers less aggressive cointercalation chemistry compared to traditional glyme cointercalation. The as-formed t-GICs with smaller interlayer spacing are highly reversible upon continuous electrochemical cycling, which informs the operation of Li^+^-THF cointercalation chemistry for extreme-condition batteries.

### Electrolyte Properties and Solvation Structure

**Figure 3:**
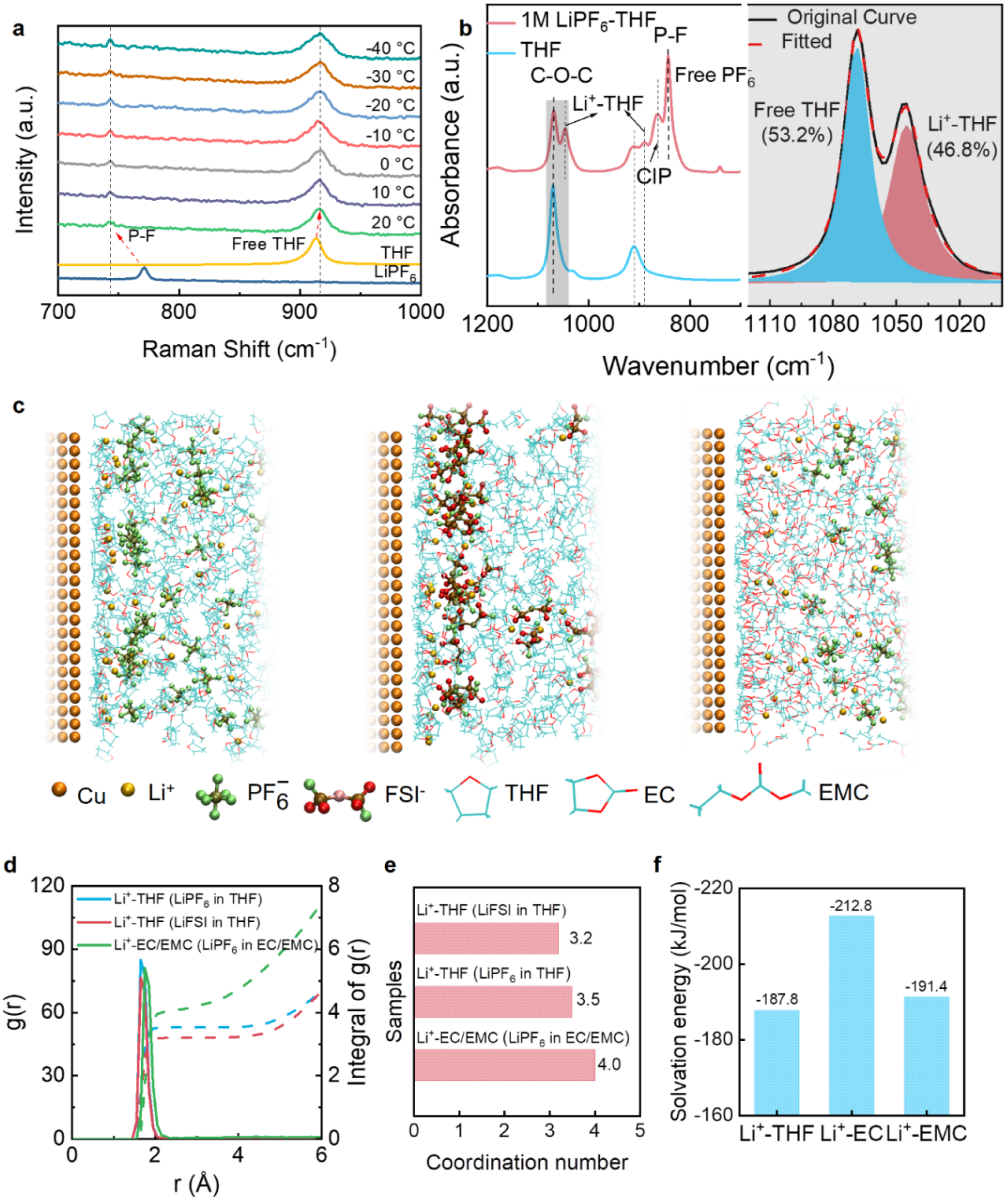
Electrolyte properties and solvation structure. a) Raman spectroscopy of the 1 M LiPF_6_-THF at different temperatures. b) FTIR spectrum of 1 M LiPF_6_-THF. c) The double-layer structure near the anode in 1 M LiPF_6_-THF (left), 1 M LiFSI-THF (middle), and 1 M LiPF_6_-EC/EMC (right) electrolytes. The surface charge on the electrode is −0.1 C m^–2^. d) The radial distribution function g(*r*) and the integrated g(*r*) between the Li^+^ in the double layer and the solvent molecules. e) The coordination number of the interfacial solvation structures. f) The solvation energy between Li^+^ and different solvent molecules.

Next, we examined the physicochemical properties and solvation structure of the 1 M LiPF_6_-THF electrolyte. The electrolyte presents excellent ion conductivity in the range of 20 to −40 °C, much higher than that of the commercial carbonate electrolyte, especially in the low-temperature region (Figure S18a). Moreover, the electrolyte remains clear with no salt precipitation and has good fluidity at −40 °C (Figure S18b). Differential scanning calorimetry (DSC) further measures that the electrolyte maintains a liquid phase even at −103 °C, highlighting subzero temperature applications (Figure S18c). Besides the ion conductivity and viscosity, the solvation effect is also crucial for ion transport in batteries as it determines the desolvation kinetics and interphase properties.^25−28^ Therefore, Raman spectroscopy and Fourier transform infrared spectroscopy (FTIR) are performed on the electrolyte. 1 M LiFSI-THF and 1 M LiPF_6_-EC/EMC are used for comparison. Figure 3a shows the Raman spectra of the 1 M LiPF_6_-THF electrolyte at different temperatures. The pure LiPF_6_ salt displays one peak at 771 cm^–1^, corresponding to the P–F stretching vibration. After dissolving in THF, the P–F peak shifts to 742 cm^–1^, indicating the strong dissociation between Li^+^ and PF_6_^–^. The THF ring breathing shifts from 913 to 916 cm^–1^ after adding LiPF_6_, indicating a weak interaction between THF and Li^+^. More importantly, these shifted peaks remain at the same position after the temperature is decreased from 20 to −40 °C, suggesting the low-temperature stability of the electrolyte. For the LiFSI-THF electrolyte, the vibration band of THF still maintains at 913 cm^–1^, indicating a weaker interaction between THF and Li^+^ (Figure S19a). Whereas the vibration band of the coordinated EC/EMC solvent is observed apparently, indicating a strong interaction between Li^+^ and EC/EMC (Figure S19b). Figure 3b presents the FTIR results of the 1 M LiPF_6_-THF electrolyte. A clear contact ion pair (CIP) peak is observed at 864 cm^–1^.^29^ The C–O–C in THF is coordinated with Li^+^ after introducing LiPF_6_. Further fitting of the peak ratio, the Li^+^-THF content accounts for 46.8%.

Classical molecular dynamics (cMD) simulations are conducted to understand the dominating solvation structure at the anode surface in the electrolyte. Simulation boxes in the cMD are constructed by sandwiching the electrolyte between two electrodes with a surface charge of ±0.1 C m^–2^. The double-layer structure near the anode in these three electrolytes is shown in Figure 3c. The solvent|cation|anion layered structure is a result of the surface adsorption of the polar solvent molecules and the repelling Coulombic force to the anions. The Li^+^-O radial distribution function g(*r*) analysis is performed by taking the first Li^+^ layer adjacent to the anode as the central ion to study the interfacial solvation structures (Figure 3d). According to the solvation number from the solvent molecules (Figure 3e), THF-based electrolytes present the interfacial solvation structures dominated by a mixture of CIP and the solvent-separated ion pair (SSIP), while it is majorly SSIP in the EC/EMC-based electrolyte. As a result, the binding energy of Li^+^-THF is smaller than that of Li^+^-EC and Li^+^-EMC (Figure 3f). Thus, Li^+^ can be readily desolvated from THF, decreasing the coordination number from Li^+^-(THF)_3.5_ to Li^+^-(THF)_1_ prior to cointercalation.

### Battery Performance

**Figure 4:**
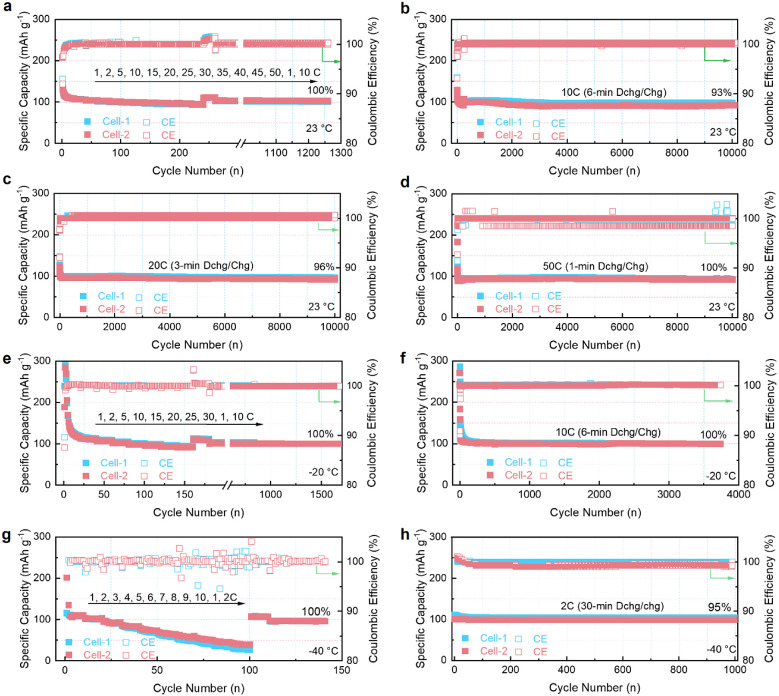
G||Li cells under extreme conditions for extended cycling. a) Rate capability of G||Li cell at the current range of 1 to 50 C (1 C = 100 mA g^–1^), and then continue to cycle at 10 C after the rate performance. b–d) Long-term cycling performance of G||Li at 10, 20, and 50 C, respectively. e) Rate capability of G||Li cell at the current range of 1 to 30 C at −20 °C, and then continue to cycle at 10 C after the rate performance. f) Long-term cycling performance of the G||Li cell at 10 C at −20 °C. g) Rate capability of G||Li cell at the current range of 1 to 10 C at −40 °C and that when it was continued to cycle at 2 C after the rate performance. h) Long-term cycling performance of G||Li cell at 2 C and −40 °C. Cell-1 and Cell-2 are repeated measurements under identical experimental conditions.

Our extensive kinetic analyses shown in Figure S20 demonstrate that D_Li_^+^_–THF_ of graphite is almost the same during cycling at 23 and −40 °C and that the Li^+^-THF storage process in graphite is similar to the surface-limited capacitive reaction allowing for fast charging. Then, the electrochemical performance of the G||Li cell in the 1 M LiPF_6_-THF electrolyte is tested at different current densities and temperatures. The rate capability of G||Li cell is shown in Figure 4a and Figure S21a, where the average reversible capacities are 110, 105, 100, 98, and 97 mAh g^–1^ at 1, 10, 20, 30, and 40 C, respectively. Even at 50 C, the reversible capacity reaches 93 mAh g^–1^, ∼85% of the capacity at 1 C, demonstrating the ultrafast kinetics of the Li^+^-THF cointercalation. After returning to 1 C, the reversible capacity maintains its original value and can be further cycled 1000 times at 10 C without capacity fading (Figure 4a). The new G||Li cell can be cycled 10 000 times at 10 C with a capacity retention of 93%, indicating the superior stability of the Li^+^-THF cointercalation (Figure 4b and Figure S21b). Figure 4c,d exhibits the ultralong cycling stability of the G||Li cell under ultrafast charging. At 20 C (3 min discharge/charge), the G||Li cell delivers a reversible capacity of 100 mAh g^–1^ and is cycled stably 10 000 times with 96% capacity retention (Figure 4c and Figure S21c). Further increasing to 50 C (1 min discharge/charge), the G||Li cell still can maintain a high reversible capacity of 92 mAh g^–1^ after 10 000 cycles, with no capacity decay compared to the initial capacity (Figure 4d and Figure S21d). The low-temperature fast charging is further demonstrated in Figure 4e–h. When cycled at −20 °C, the G||Li cell delivers reversible capacities of 111, 101, 95, and 91 mAh g^–1^ at 1, 10, 20, and 30 C, respectively, barely different from the room-temperature capacity (Figure 4e and Figure S21e). When cycled at 10 C, the G||Li cell maintains superior cycling stability with a reversible capacity of 100 mAh g^–1^ for 3,700 cycles at −20 °C, consistent with the initial capacity (Figure 4f and Figure S21f). Further cooling down to −40 °C, the reversible capacity at 1, 2, 5, and 10 C are 110, 102, 75, and 40 mAh g^–1^, respectively (Figure 4g and Figure S21g). Although the rate capability decreases, the reversible capacities at 1 and 2 C are almost the same as that at room temperature, highlighting the excellent low-temperature fast charging performance (Figure 4g). Moreover, the G||Li cell shows excellent cycling stability for 1000 cycles at 2 C with 100% capacity retention (Figure 4h and Figure S21h). The electrochemical performance of the G||Li cell is further summarized in Figure S21i. Such ultrafast charging performance at room and low temperatures has never been reported in graphite anodes elsewhere (Figure S21j and Table S1). The morphological evolution of the cycled graphite is characterized by SEM images. After 100 cycles, severe expansion of the graphite layer can be clearly observed in Figure S21, but the overall structure is still well preserved without obvious exfoliation, showing its high mechanical stability. Moreover, there is no Li plating and “dead Li” formation on the graphite surface or in the separator (Figure S22a-c). The EDS mapping shows that C, F, P, and O elements are well-distributed on the surface of the cycled graphite, and the C content accounts for 91.7%, indicating that the electrolyte is stable without excessive decomposition (Figure S22e).

**Figure 5:**
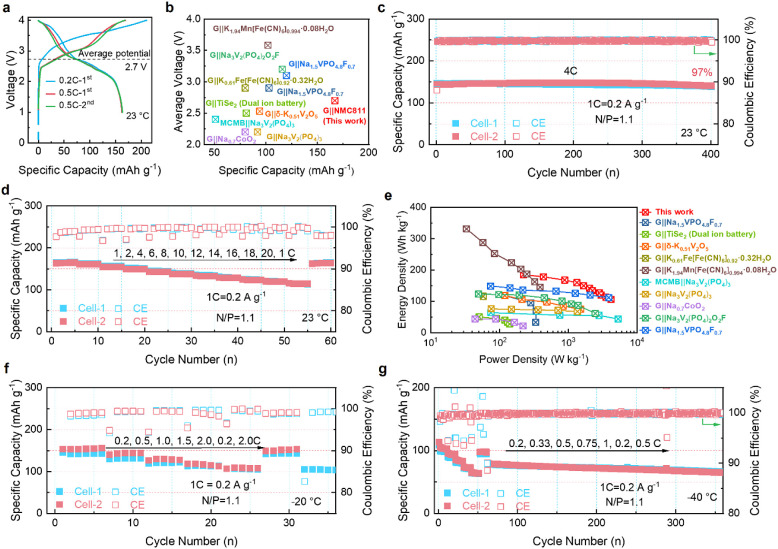
High power density and high energy density Li-ion full cells. a) Voltage profiles of the G||NMC811 full cell at 0.2 and 0.5 C (1C = 0.2 A g^–1^). b) Comparison of average voltage and capacity of the G||NMC811 full cell (based on the cathode) with recently reported Na-/K-based full cells.^6,21,30 −37^ c) Long-term cycling performance of the G||NMC811 full cell at 4 C. Full cell operation at 23 °C. d) Rate capability of the G||NMC811 full cell at the current range of 1 to 20 C. Full cell operation at 23 °C. e) Comparison of energy density and power density of the G||NMC811 full cell (based on the total mass of cathode and anode) with recently reported Na-/K-based full cell.^6,21,30 −37^ f) Rate capability of the G||NMC811 cell at the current range of 0.2 to 2 C. Full cell operation at −20 °C. g) Rate capability of the G||NMC811 cell at the current range of 0.2 to 1 C and that when it was continued to cycle at 0.5 C after the rate performance. Full cell operation at −40 °C. Cell-1 and Cell-2 are repeated measurements under identical experimental conditions.

To evaluate the fast-charging and low-temperature performance of the graphite anode in Li-ion full cells, NMC811 is selected as the cathode for full cells. No precycled process is required since the graphite anode has high ICEs in the 1 M LiPF_6_-THF electrolyte, which is superior to the recently reported cointercalation cells (Table S2). Three formation cycles are performed with one cycle at 0.2 C and then two cycles at 0.5 C (1C = 0.2 A g^–1^) (Figure 5a). The full cell delivers a capacity of 163 mA g^–1^ based on the mass of the cathode, with an average voltage of ∼2.7 V at 0.5 C. The capacity is much higher than those of Na-based or K-based cells (Figure 5b). As shown in Figure S23, the full cell delivers an initial capacity of 133 mAh g^–1^ at 4 C when only a constant current charging (CCC) is performed. After 800 cycles, the full cell retains a reversible capacity of 123 mAh g^–1^, with an extremely low capacity decay rate of 0.009% per cycle, validating the high stability of NMC811 and graphite in the 1 M LiPF_6_-THF electrolyte. When a constant voltage charging (CVC) procedure is added to the CCC step for a complete charging process of 15 min. The full cell delivers a high initial capacity of 146 mAh g^–1^ at 4 C with a capacity retention of 97% after 400 cycles (Figure 5c). Figure 5d displays the rate capability of the full cell at current densities ranging from 1 to 20 C (1.5 min charging process), where the reversible capacities are 167, 163, 157, 151, 145, 139, 134, 129, 125, 120, and 114 mAh g^–1^, respectively (Figure S24a). Approximately 68% of the reversible capacity is retained even with a 20-fold increase in current density from 1 to 20 C. The high rate capability renders a remarkably high power density of 4180 W kg^–1^ with an energy density of 106 Wh kg^–1^ (Figure 5e, based on the total mass of the cathode and anode), much higher than tha of Na-based and K-based cells (Table S2). The low temperature (and fast charging) performance is further tested. The full cell also shows excellent rate performance at −20 °C, delivering 142, 131, 120, 113, and 105 mAh g^–1^ at 0.2, 0.5, 1.0, 1.5, and 2.0 C, respectively (Figure 5f and Figure S24b). An impressive capacity (134 mA g^–1^) and stability (∼92%) are also obtained after 150 cycles at 1 C (Figure S25a, S25b). Even at −40 °C, the full cell using the 1 M LiPF_6_-THF electrolyte exhibits excellent rate performance (Figure 5g and Figure S24c). As shown in Figure 5g, the reversible capacities are determined to be 101, 91, 80, 69, and 61 mAh g^–1^ at 0.2, 0.333, 0.5, 0.75, and 1 C, respectively. Upon continuing cycling at 0.2 C, the full cell maintains 83% of its initial capacity after 150 cycles (Figure S25c-d). Switching cycling to 0.5 C, the full cell shows 84% capacity retention after 300 cycles (Figure 5g and Figure S24c). Such excellent low-temperature performance has never been reported in Li-ion full cells (Table S3).

## Conclusion

In this work, we discover a new intercalation chemistry superior to traditional glyme cointercalation. t-GICs are in situ synthesized in the 1 M LiPF_6_-THF electrolyte via a controllable chemical reaction between b-GIC and THF during battery cycling, which enables reversible, rapid Li^+^-THF cointercalation into graphite. The operando X-ray and electrochemical analyses further confirmed this spontaneous synthesis. Compared to linear ether, cyclic ether has a weak interaction with Li^+^ due to the steric hindrance, which allows for facile partial desolvation of Li^+^-(THF)_3.5_ to yield Li^+^-(THF)_1_ for rapid cointercalation. Our electrolyte has also allowed battery reactions in individual graphite particles to proceed synchronously even at fast charging. As a result, the graphite anode displays an ultralong lifespan of over 10 000 cycles with negligible capacity decay, 1 min fast charging, and unprecedented low-temperature performance (including fast charging) with no lithium dendrite formation. Coupled with the NMC cathode, the full cell displays an excellent balance between energy and power in the Ragone plot and outstanding cycling stability for 800 cycles within 15 min charging. Even at −40 °C, the excellent rate capability can be achieved, with 100 mAh g^–1^ at 0.1 C, 90 mAh g^–1^ at 0.33 C, 80 mAh g^–1^ at 0.5 C, and 60 mAh g^–1^ at 1 C, respectively. Graphite anodes with such high-power capability in LIBs have never been reported elsewhere. THF is a low-cost, mass-produced chemical, making 1 M LiPF_6_-THF an attractive electrolyte for commercialization. Last but not least, our work provides new insights into GIC synthesis and opens the door to designing beyond graphite intercalation anodes for fast charging and low-temperature operations.

## Data Availability

All relevant data in the article are available from the corresponding author upon reasonable request.

## Supporting Information Available

Operando optical microscopy of graphite during cycling (MP4)
Operando coherent X-ray multicrystal diffraction (MP4)
Materials and methods, electrochemical measurements, Operando synchrotron XRD patterns, Operando CMCD patterns, Raman spectra, SEM images, and TEM images (PDF)
