# Supplementary material for: Solvent-Mediated, Reversible Ternary Graphite Intercalation Compounds for Extreme-Condition Li-Ion Batteries
Source: J Am Chem Soc. 2024 Jun 7;146(24):16764–74. doi: 10.1021/jacs.4c04594 (PMC11191681; doi:10.1021/jacs.4c04594)
Supplement: Supplementary file 3 — ja4c04594_si_003.pdf [file ja4c04594_si_003.pdf]

Supporting information

Solvent-mediated, reversible ternary graphite intercalation compounds for extreme-condition Li-ion batteries

Lei Tao[1*&], Dawei Xia[1&], Poom Sittisomwong[2], Hanrui Zhang[3], Jianwei Lai[3], Sooyeon Hwang[4], Tianyi Li[5], Bingyuan Ma[2], Anyang Hu[1], Jungki Min[1], Dong Hou[1], Sameep Rajubhai Shah[6], Kejie Zhao[6], Guang Yang[7], Hua Zhou[5], Luxi Li[5], Peng Bai[2], Feifei Shi[3], Feng Lin[1,8*]

[1] Department of Chemistry, Virginia Tech, Blacksburg, VA 24061, USA

[2] Department of Energy, Environment & Chemical Engineering, Washington University in St. Louis, St. Louis, MO 63130, USA

[3] Department of Energy and Mineral Engineering, The Pennsylvania State University, University Park, PA 16802, USA

[4] Center for Functional Nanomaterials, Brookhaven National Laboratory, Upton, NY 11973, USA

[5] X-Ray Science Division, Argonne National Laboratory, Lemont, IL 60439, USA

[6]Mechanical Engineering, Purdue University, West Lafayette, IN 47907, USA

[7] Chemical Sciences Division, Oak Ridge National Laboratory, Oak Ridge TN 37830, USA

[8] Department of Materials Science and Engineering, Virginia Tech, Blacksburg, VA 24061, USA

[&]L. Tao and D. Xia contributed equally to this work.

[*]Corresponding: taolei@vt.edu; fenglin@vt.edu.

1. Experimental section

1.1. Materials

Superior graphite (G), natural graphite (NG), Mesocarbon microbeads (MCMB), and NMC811 are provided by the Cell Analysis, Modeling, and Prototyping (CAMP) Facility in Argonne National Lab. Lithium hexafluorophosphate ($LiPF_6$) and Lithium Bis (fluorosulfonyl)imide (LiFSI) are purchased from Gotion Inc. Tetrahydrofuran (THF) solvent is purchased from Sigma Aldrich (anhydrous, ≥ 99%, inhibitor-free). Li metal foils are purchased from China Energy Lithium Co., Ltd. Before any electrolyte preparation, the solvents are treated with an activated molecular sieve to remove traces of moisture. The salt is dried in the glovebox before use. LP57, in the formula of 1 M $LiPF_6$-EC/EMC (with a weight ratio of 3/7), is purchased from Gotion Inc. (battery grade).

1.2. Electrochemical measurements

G||Li cell assembly and testing. The G anode is prepared by mixing G and sodium alginate (at a mass ratio of 9:1) in the DI water, stirring to form a homogenous slurry, and casting on Cu foil. The prepared G anode is punched into φ12.7 mm disks and dried overnight in a vacuum oven at 120 °C. The mass loading of the active material is close to 2.5 mg/cm^2. The electrolyte is a 1 M $LiPF_6$ dissolved into THF solvent. Two other electrolytes: 1 M LiFSI-THF and 1 M $LiPF_6$-EC/EMC, are used for comparison. The G||Li cell is assembled from the G anode, 60 μL electrolyte, GF/A separator, and Li foil in an Ar-filled glovebox with H_2O and O_2 level ≤ 0.1 ppm. The cell is tested at room and low temperatures using a NEWARE battery test system in a voltage range of 0-3V (vs. Li/Li^+).

Galvanostatic intermittent titration technique (GITT). Before the GITT test, the G||Li cell is precycled at 1C (1C=0.1A g^{-1}) for 5 cycles. Then, the G||Li cell is discharged/charged at 1C with a current pulse duration of 3 mins and an interval time of 4 h.

Cyclic voltammetry (CV). The CV measurements are performed between 0-3 V (vs. Li^+/Li) by using a single-channel potentiostat (Princeton VersaSTAT 4) at scanning rates from 0.2 mV s^{-1} to 1.0 mV s^{-1} at room temperature. The relationship between the peak current (i) and scan rate (v) followed Equations (1), where the b value can be calculated based on Equations (2).

$$i = av^b \tag{1}$$

$$log(i) = blog(v) + log(a) \tag{2}$$

Typically, the b value that is close to 0.5 indicates diffusion-dominated storage, while the value close to 1.0 represents surface-controlled capacitive storage. The contribution of these two behaviors can be calculated following Equation (3).

$$i(V) = k_1 v + k_2 v^{1/2} \quad (3)$$

Electrochemical impedance spectroscopy (EIS). The EIS measurements are tested in the frequency range of 10 mHz to 500 kHz at -20 to 20 °C. The activation energies can be calculated based on the Arrhenius equations (4) by fitting the temperature-dependent resistances:

$$\frac{1}{R(ct,\ SEI)} = A_0 e^{-\frac{Ea}{RT}} \quad (4)$$

where A_0 is the pre-exponential constant, R refers to the standard gas constant, and E_a represents the activation energy.

Ionic conductivity measurement. Stainless steel (SS) symmetric cells are assembled, sandwiching the Celgard 2325 (1 layer) with a thickness of 25 µm. The diameter of SS is 15.8 mm. The electrolytes are flooded, and the cells are rested overnight. EIS is recorded for the cell with one repetition. Here the frequency range is 1 Hz to 1MHz. The ionic conductivity is calculated using the following equation:

$$\sigma = \frac{d}{R_b \times S}$$

where d is the thickness of Celgard, and Rb is the bulk resistance derived from Nyquist plots. S is the surface area of the SS.

G||NMC811 cell assembly and testing. The cathode is prepared by mixing NMC811 active material, carbon black, and PVDF (at a mass ratio of 9:0.5:0.5) in the NMP solvent to form a slurry and then doctor-blade casting on a carbon-coated aluminum foil. The prepared cathode is punched into 10 mm diameter disks and dried overnight in a vacuum oven at 120 °C. The mass loading of the NMC811 cathode is 3.0~5.0 mg/cm^2. The full cell is assembled using an NMC811 cathode, 60 µL electrolyte (1M $LiPF_6$-THF), GF/A separator, and the fresh G anode. The full cell is tested in a voltage range of 1-4 V (vs. Li^+/Li) at room and low temperatures.

1.3. Characterization

G and cycled G morphology are characterized using a scanning electron microscope (FESEM, LEO 1550) and high-resolution transmission electron microscopy (HRTEM, JEOL JEM 2100). The chemical properties of the cycled G are quantitatively characterized by X-ray photoelectron spectroscopy (XPS, PHI VersaProbe III) using a monochromatic Al K-alpha X-ray source (1486.6

eV). The SEI films of the G after cycling in different electrolytes are observed at liquid nitrogen temperature with FEI Talos F200X and Gatan cryo-transfer holder. For TEM preparation, the cycled G||Li cell was disassembled in the glove box, and the G electrode was peeled off from the current collector and washed several times with the THF solvent to remove the residual salts. Then, the washed G electrode is dispersed in the THF solvent and dropped onto the Cu TEM grid. To avoid contamination or exposure to air, the samples are sealed, transported, and loaded into the cryo-TEM holder under liquid nitrogen conditions. Fourier-transform infrared spectroscopy (FTIR) spectra of the G and cycled G are recorded using a Nexus 670 FTIR spectrometer. Raman measurements of the G, cycled G, and electrolytes are performed on a Horiba Jobin-Yvon using a 532 nm laser. X-ray diffraction (XRD) spectra of the G and cycled G are collected from 20 ° to 90 ° using an automated diffractometer (D8 Advance, Bruker, Karlsruhe, Germany) with Cu Ka radiation at room temperature. Differential scanning calorimetry (DSC, Netzsch 200F3) was performed to measure the melting point of the electrolyte from -160 to 23°C at a temperature ramp rate of 5°C/min. Thermogravimetric analysis (TGA) was performed to measure the mass loss of lithiated graphite (THF-based) from 23 to 600 °C with a heating rate of 5°C/min. To better estimate the number of THF molecules participating in the reversible co-intercalation, we calculated the mass change of the graphite electrode at different discharge states. All graphite electrodes have the same mass loading of 7mg/cm^2. To eliminate the influence of residual solvent and salt, the disassembled graphite electrode is thoroughly cleaned and soaked in the THF solvent for 30 min. Then, it is dried at 60°C for 12h. The whole process was operated in an Ar-filled glovebox and three repeated tests were performed.

Operando synchrotron XRD. The *operando* XRD is performed by synchrotron X-rays (λ=0.1173 Å) in a transmission mode in Advanced Photon Source (APS Beamline 11-ID-C, APS, Argonne National Lab). The beamline is optimized for high-energy XRD at 105.7 keV. The cell is assembled using 2032-coin cells with a 3 mm diameter hole sealed by Kapton tape in the center. Before refinement, the sample-to-detector distance, detector tilt angles, and instrumental parameters are calibrated by a standard sample of CeO_2. The 2D diffraction rings are integrated into intensity vs 2 theta data by using the Fit2D software. The XRD data is refined using Rietveld refinement in the FullProf Suite software. The average size is calculated from the full width at half maximum of the diffraction peaks according to the size and microstrain model.

Operando coherent X-ray multicrystal diffraction (CMCD). The in-situ coherent X-ray multicrystal diffraction (CMCD) experiment was performed at the 12-ID-D beamline of the Advanced Photon Source, Argonne National Laboratory. The X-rays were selected by a double-crystal monochromator at 12 keV (or 1.033167 Å). The X-ray beam was focused by compound reflective lenses (CRLs) to 2um spot size, and the sample was located downstream of the focus spot with a 50-60 um spot size to illuminate enough particles. An Eiger 500K direct photon counting detector was located 0.5 m downstream from the sample to collect the diffraction patterns repeatedly under the sample operando conditions.

1.4. Simulation

cMD Simulation: The classical MD simulation was performed using LAMMPS[1] at constant volume and temperature (NVT) to the electrolyte-electrode nano-slit geometry[2] with two 40×40×20 $Å^3$ Cu electrodes in an FCC lattice sandwiching the 40×40×100 $Å^3$ electrolyte. The model was constructed based on the OPLSAA force field[3] where the partial charges of EC, EMC, and THF molecules are optimized using Gaussian 16 B3LYP and 6-311++G(d,p) basis set, then the partial charges are calculated by using Merz–Kollman (MK).[4–8] The FSI^- anion was constructed based on the CL&P force field.[9] The geometric combination rule for the Lennard-Jones parameters between different atom types was adopted. Surface charge was applied by placing partial charge on the first layer of Cu atoms. Equilibration was performed at 1 fs time step for 10 ns at 400 K and then another 10 ns at room temperature without surface charge, followed by 20 ns simulation with the surface charge of ±0.05 then ±0.1 C m^{-2}. The final 2 ns of ±0.1 C m^{-2} surface charge was used for data collection. The initial configuration was generated by the open-source software Moltemplate and PACKMOL.[10,11] RDF analysis and visualization were performed using VMD.[12]

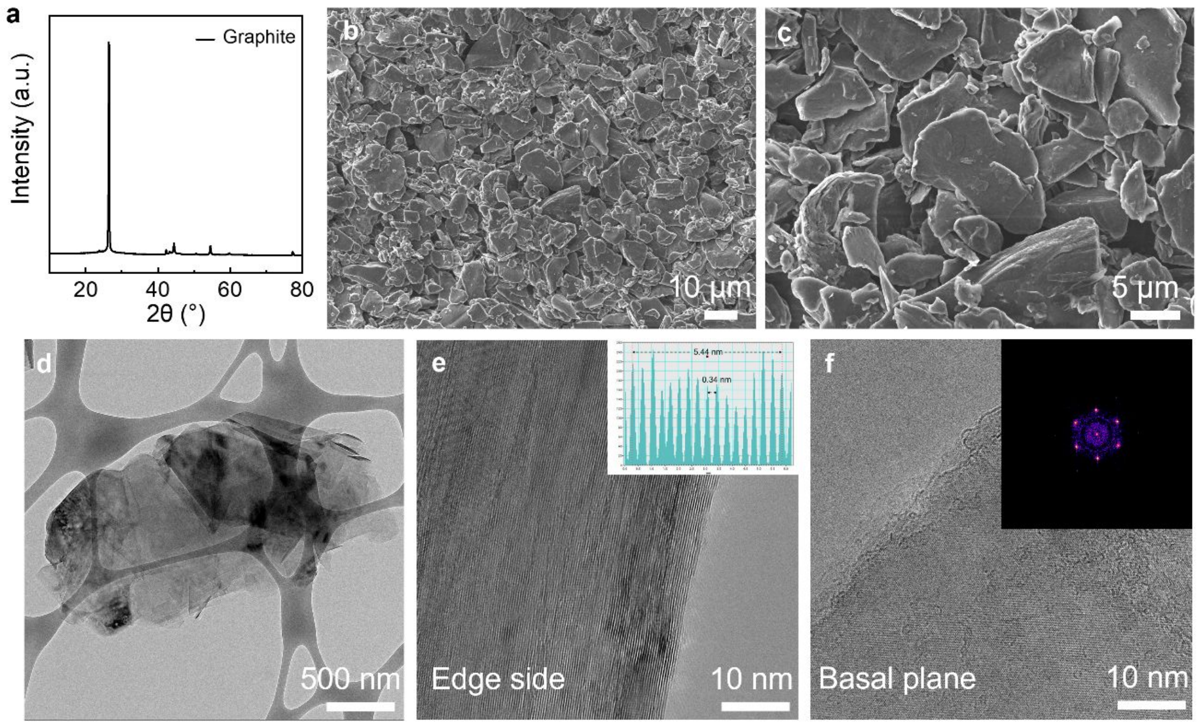

Fig. S1| Structure and morphology of the natural graphite. **a**, XRD pattern of the pristine graphite. **b-c**, SEM images of the pristine graphite at different magnifications. TEM images of the pristine graphite: **d**, low magnification; **e**, HRTEM image of the edge side; **f**, HRTEM image of the basal plane.

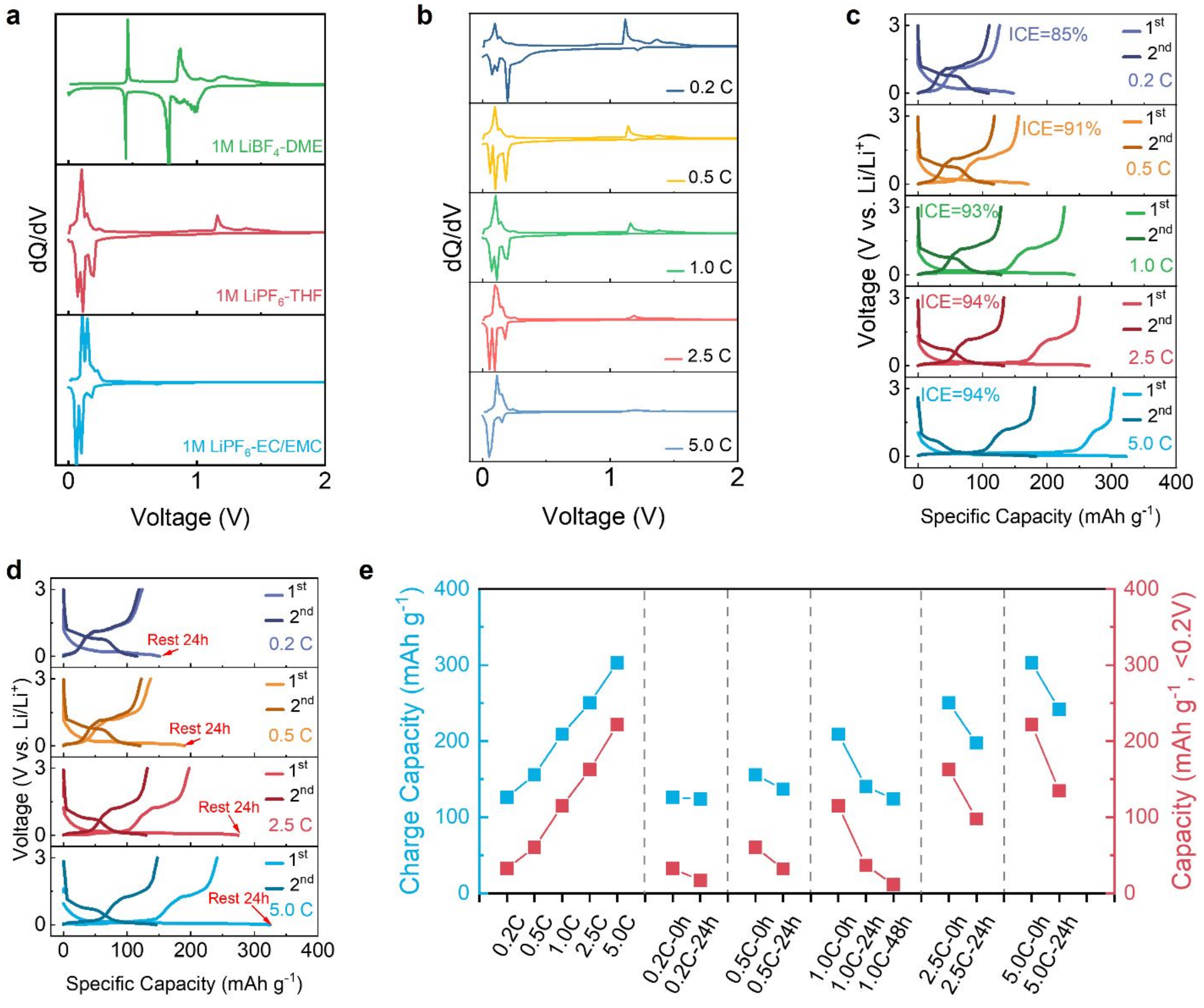

Fig. S2| a, *dQ/dV* curves of graphite electrode cycled in the 1M $LiPF_6$-EC/EMC, 1M $LiPF_6$-THF, and 1M $LiBF_4$-DME electrolyte, respectively. **b**, *dQ/dV* curves of graphite electrode cycled in the 1M $LiPF_6$-THF electrolyte at the current range of 0.2 to 5C. **c**, Voltage profiles of graphite electrode cycled at the current range of 0.2 to 5C. **d**, The discharge-charge curves of graphite anode cycled in the 1M $LiPF_6$-THF electrolyte with a resting step of 24h after the 1st lithiation. **e**, The capacities of graphite anode in the 1M $LiPF_6$-THF electrolyte at the different currents and the rest time are summarized.

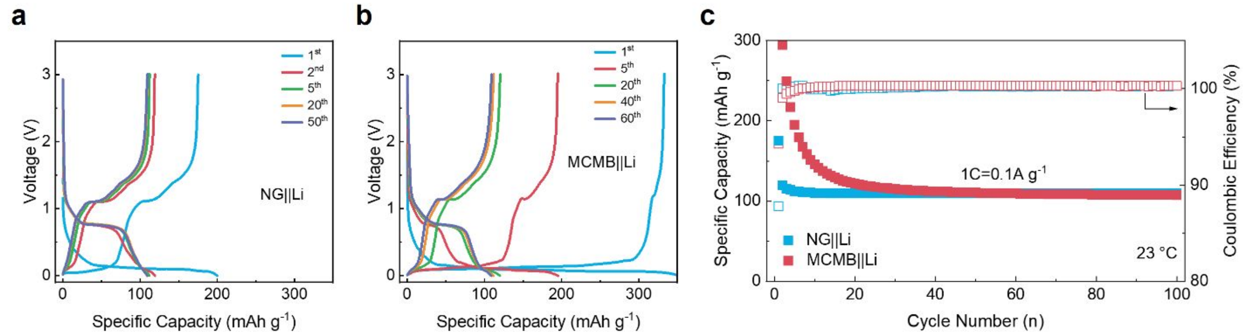

Fig. S3| Different types of graphite materials cycled in the 1M $LiPF_6$-THF electrolyte. **a**, voltage profiles of the NG||Li cell at selected cycles. **b**, voltage profiles of the MCMB||Li cell at selected cycles. **c**, corresponding long-term cycling. Mesocarbon microbeads (MCMB) and nature graphite (NG). These results show that MCMB requires more cycle numbers to achieve the stabilized capacity (complete utilization of co-intercalation without the existence of b-GIC).

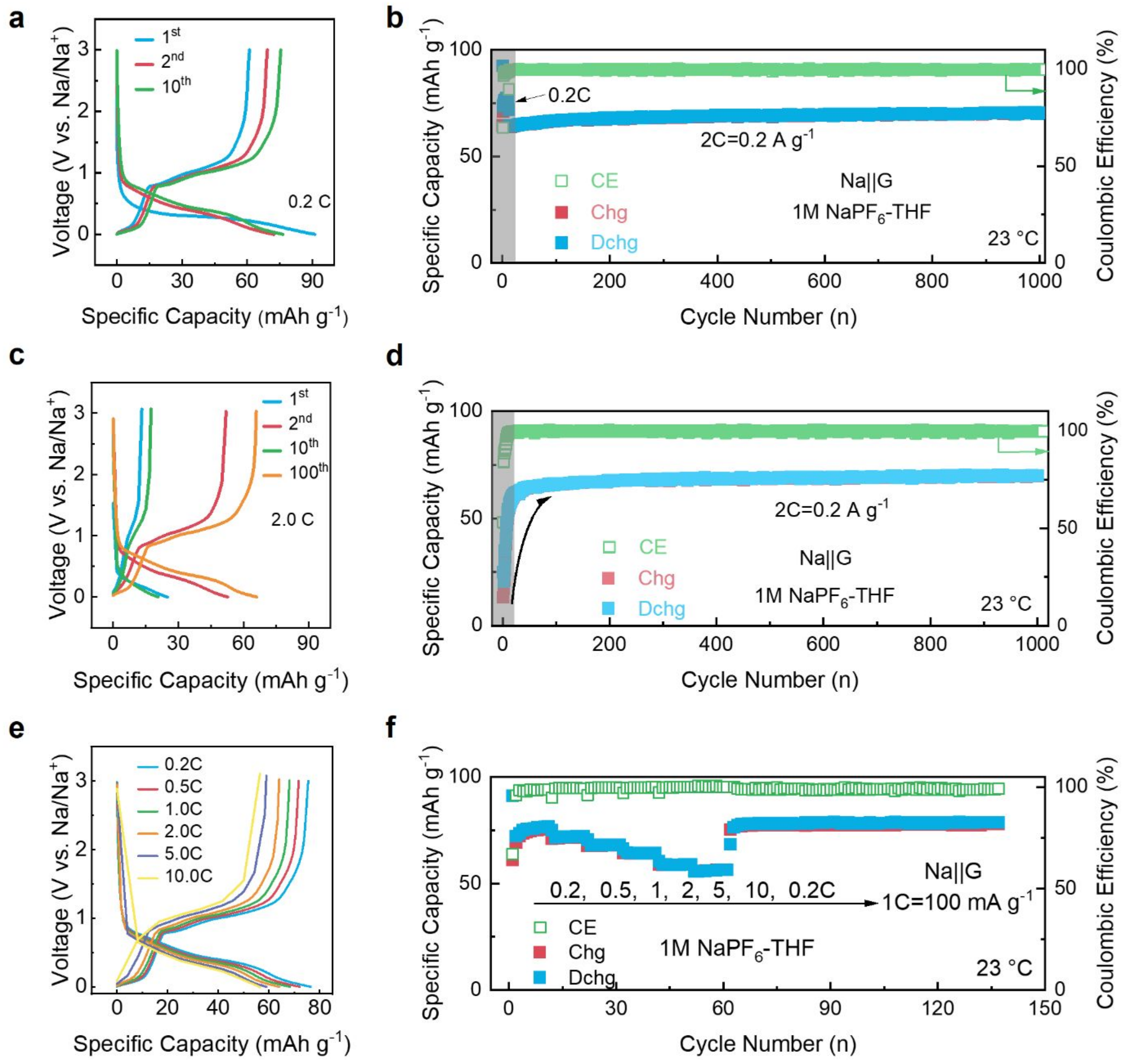

Fig. S4| a, Voltage profiles of graphite anode cycled in the 1M $NaPF_6$-THF electrolyte at 0.2 C (formation cycles), and **b,** continue to cycle at 2 C for 1000 cycles. **c**, Voltage profiles of graphite anode cycled in the 1M $NaPF_6$-THF electrolyte at 2.0 C (without formation cycles). **d**, Long-term cycling performance of graphite anode at 2 C. **e**, Voltage profiles of graphite anode cycled in the 1M $NaPF_6$-THF electrolyte at the current range of 0.2 to10.0 C. **f**, Rate performance of graphite anode cycled in the 1M $NaPF_6$-THF electrolyte.

The initial discharge-charge curve at 2 C shows that Na ions cannot intercalate into the graphite interlayer without a chemical reaction with THF. The curve is consistent with that of graphite anode cycled in 1M $NaClO_4$-EC/EMC electrolyte[13]. After several cycles, the chemical reaction

happens, and Na^+-THF is co-intercalated into the graphite interlayer with a capacity of 70 mAh g^{-1}. When we cycled at 0.2 C, the low current gave more time for the chemical reaction, so co-intercalation occurred in the first cycle with a capacity of 76 mAh g^{-1}.

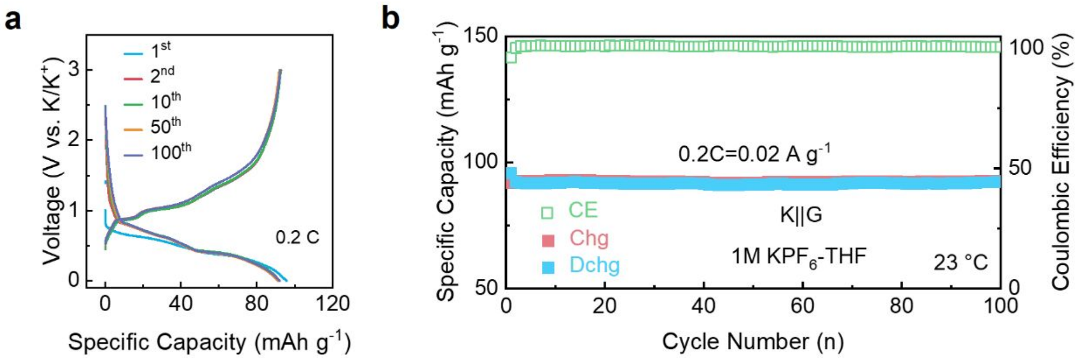

Fig. S5| a, Voltage profiles of graphite anode cycled in the 1M KPF_6-THF electrolyte at 0.2 C, and **b**, corresponding cycling performance.

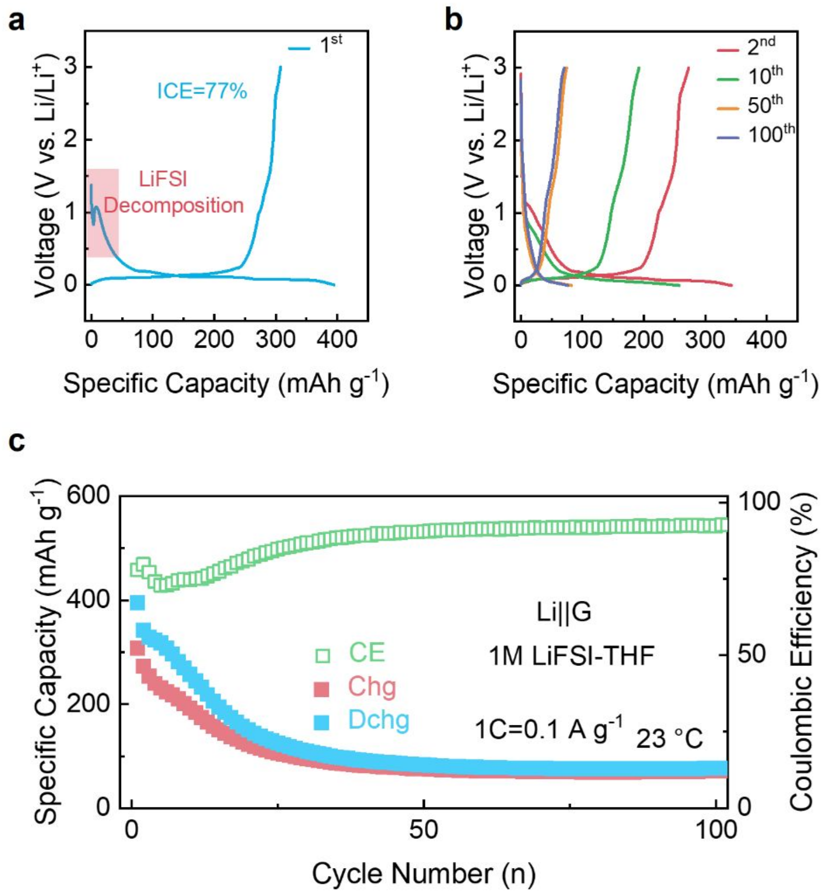

Fig. S6| a-b, The initial discharge-charge and subsequent curves of graphite anode cycled in the 1M LiFSI-THF electrolyte at 1C. **c**, Corresponding cycling performance.

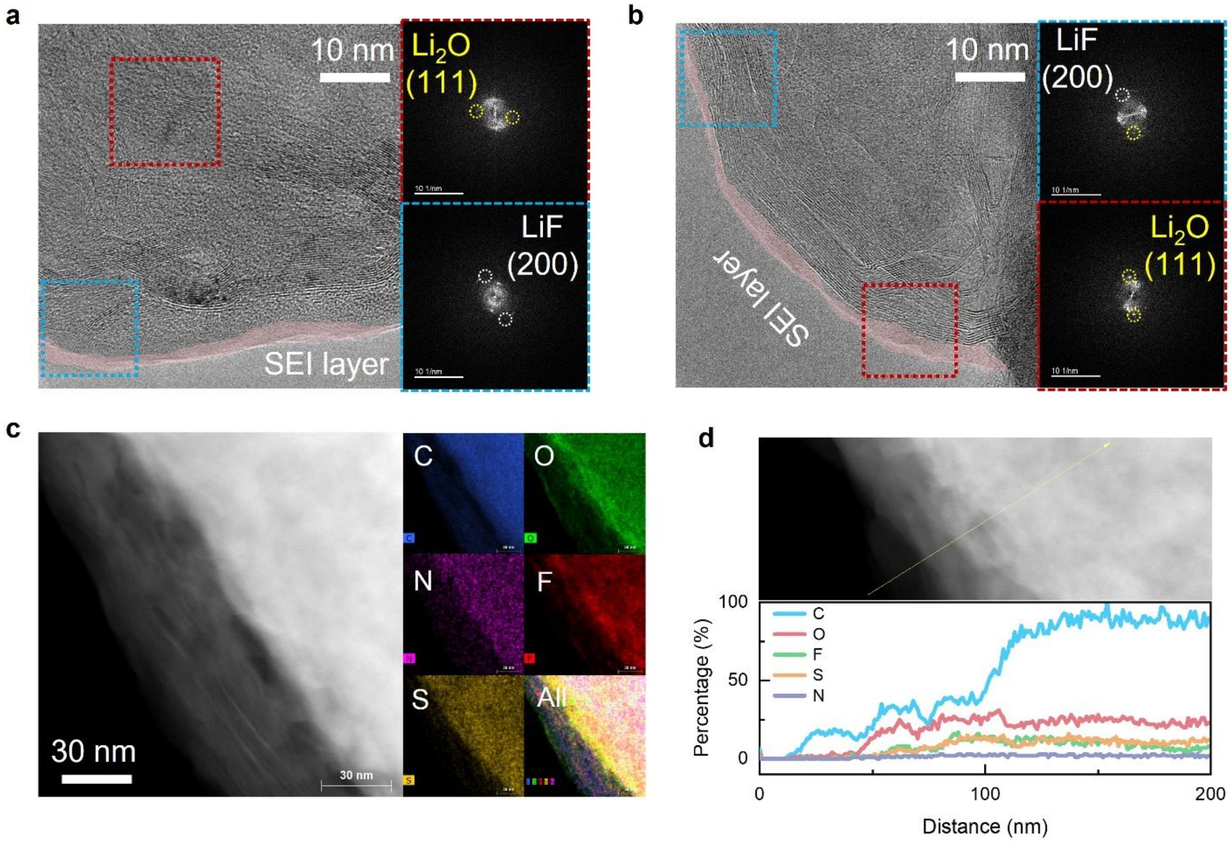

Fig. S7| a-b, Cryo-TEM images of graphite anode in the 1M LiFSI-THF electrolyte for 10 cycles. **c**, STEM-EDS elemental mapping of C, O, N, F, S, F, and all elements overlaid in one image. **d**, Line scan of the element content of the cycled graphite from surface to bulk.

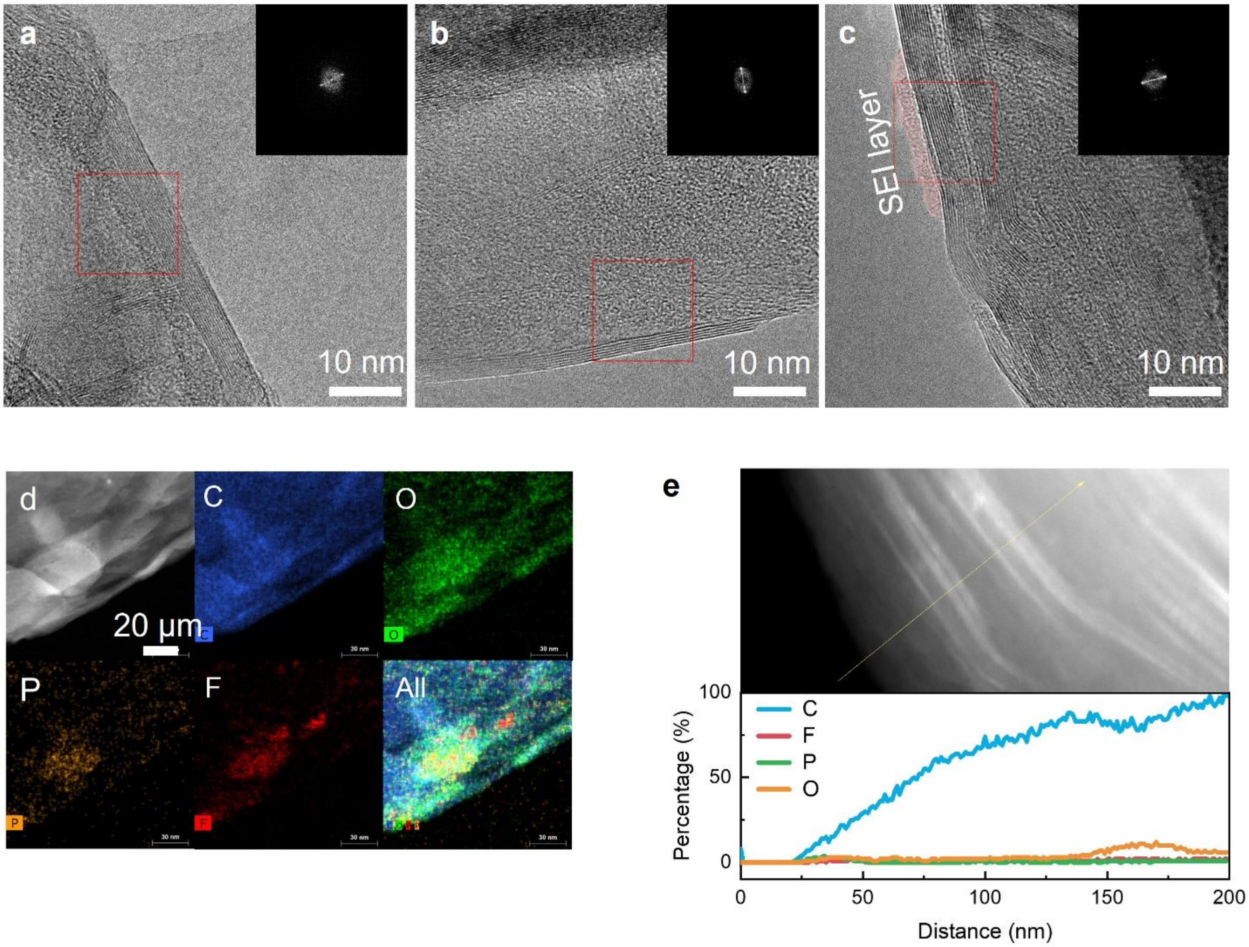

Fig. S8| a-c, Cryo-TEM images of graphite anode in the 1M $LiPF_6$-THF electrolyte for 10 cycles. **d**, STEM-EDS elemental mapping of C, O, N, F, S, F, and all elements overlaid in one image. **e**, Line scan of the element content of the cycled graphite from surface to bulk.

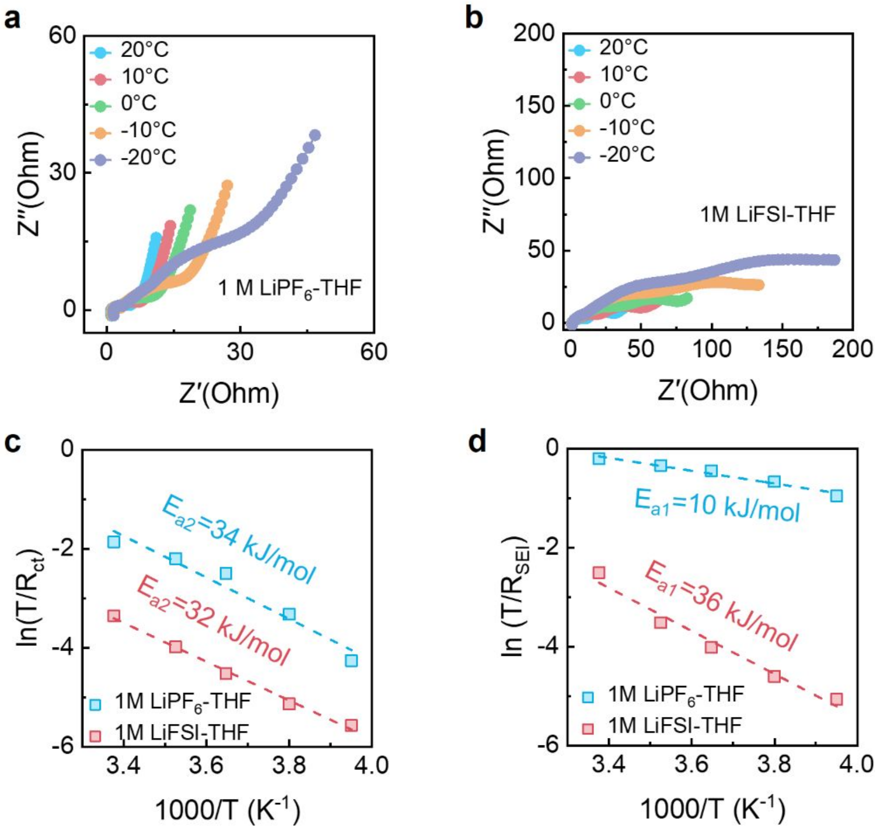

Fig. S9| Temperature-dependent electrochemical impedance spectroscopy of the G||Li cell in the: **a**, 1M $LiPF_6$-THF electrolyte; **b**, 1M LiFSI-THF electrolyte. **c-d,** Arrhenius plots of the temperature dependence of EIS resistance. The desolvation energy is known as the charge transfer process.

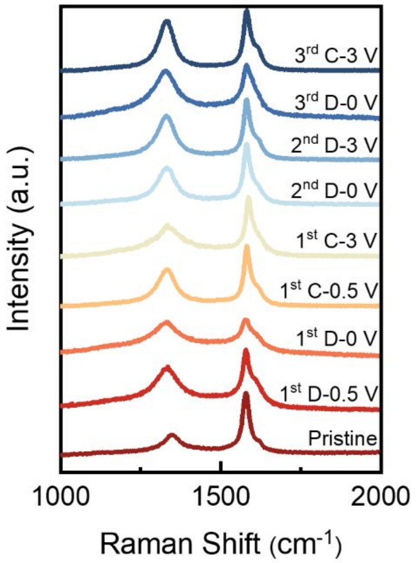

Fig. S10| The *ex situ* Raman spectroscopy of graphite anode cycled in the 1M $LiPF_6$-THF electrolyte at 1C for 3 cycles.

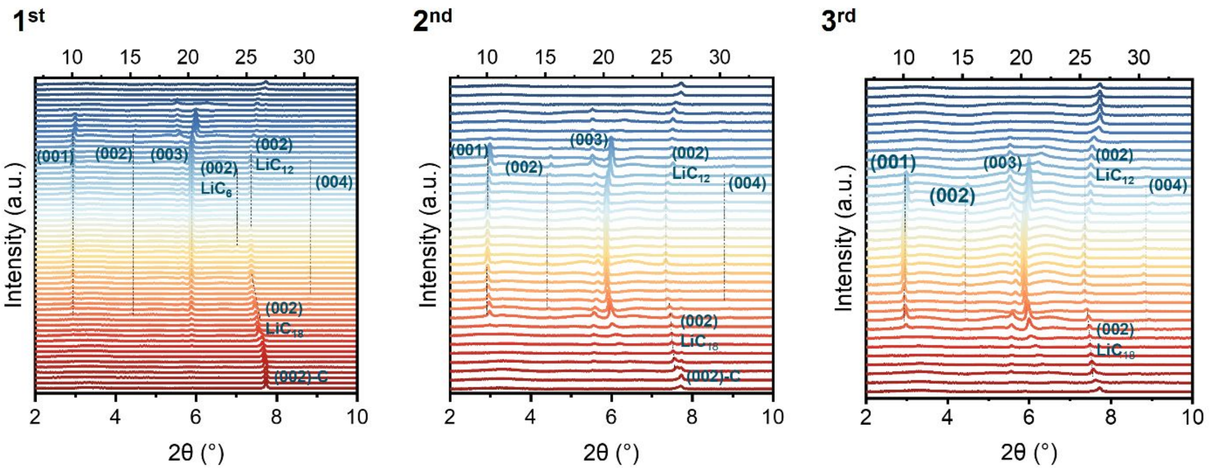

Fig. S11| Synchrotron *operando* XRD patterns of the G||Li cell at 1C for 3 cycles.

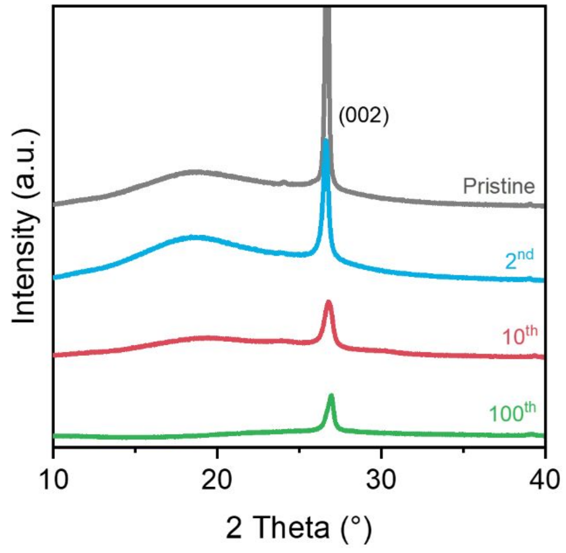

Fig. S12| XRD patterns of graphite anode cycled in the 1M $LiPF_6$-THF electrolyte at 1C for 2, 10, and 100 cycles.

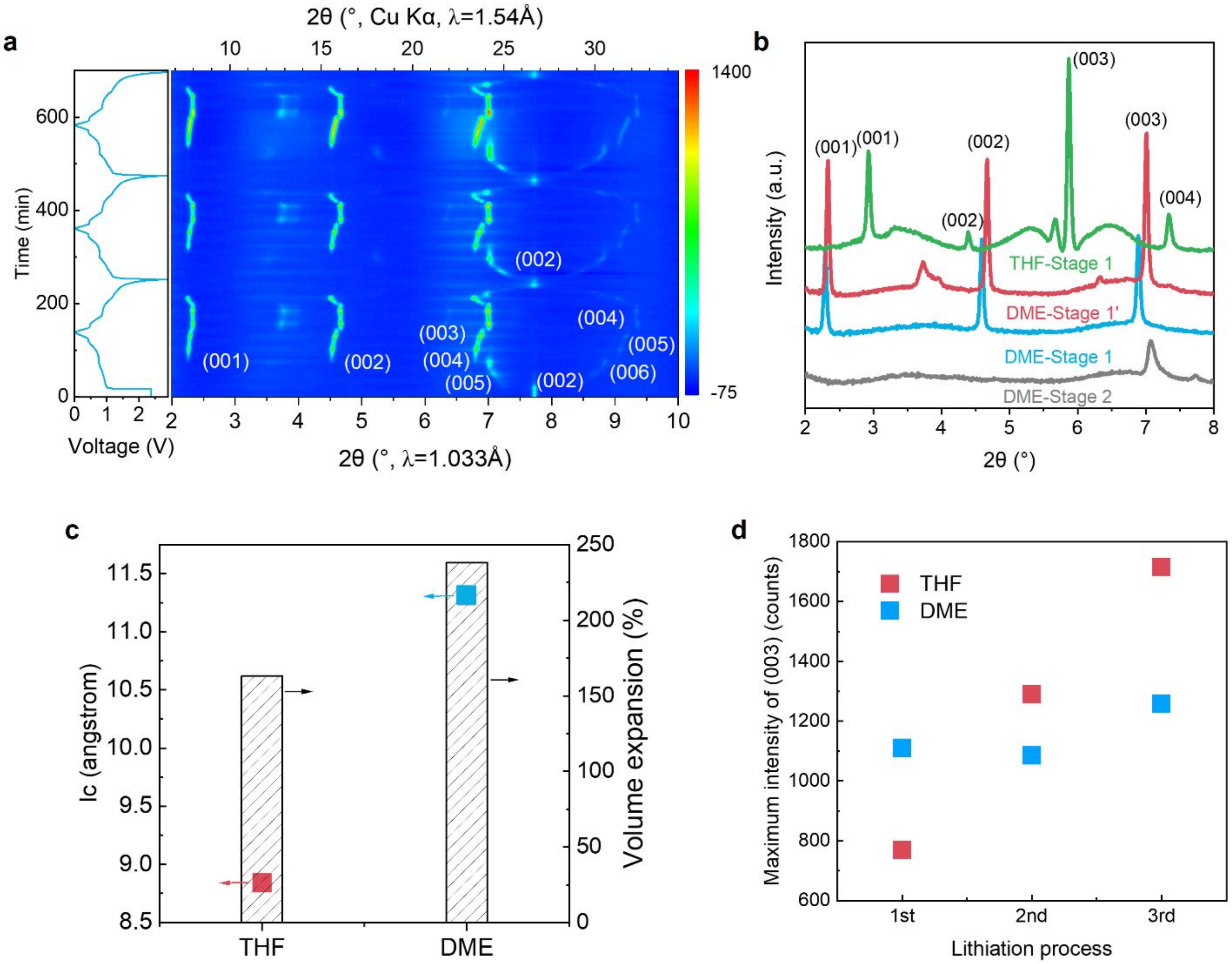

Fig. S13| a, Galvanostatic charge-discharge curves and synchrotron *operando* XRD patterns of the G||Li cell cycled in the 1M $LiBF_4$-DME electrolyte at 1C for 3 cycles. **b**, the selected patterns from the *operando* XRD measurements cycled in two different electrolytes at the discharge stages. **c**, corresponding interlayer spacing Ic and volume expansion of graphite after Li^+-solvent co-intercalation. Stage 1 GIC based on THF and DME are used for calculation. **d**, the evolution of the (003) peak intensity of different t-GICs during the first 3 cycles.

Figure S13a shows the *operando* synchrotron XRD patterns of the graphite electrode cycled in the 1M $LiBF_4$-DME electrolyte during the first three cycles. Before discharge, only one diffraction peak is located at 2θ~26.7°, corresponding to the (002) peak of graphite. Upon the initial discharge, the intensity of the (002) peak decreases and then it splits into two peaks, i.e., (005) and (006), indicating the formation of regularly ordered high-stage GICs. As the discharge continues, the (002) peak disappears, while the two new peaks shift to lower and higher angles ((004), (005)),

respectively. Finally, the two peaks shift to 2θ~24.2° and 32.2°, and the (002) peak is found at 2θ~16.1°, indicating the formation of Li^+-DME t-GICs. Such t-DME-GIC formation is completely different from that of t-THF-GIC (Figure 2a). The 2nd and 3rd cycles display similar features to the 1st cycle, suggesting the good structural reversibility of graphite during the initial Li^+-DME co-intercalation.

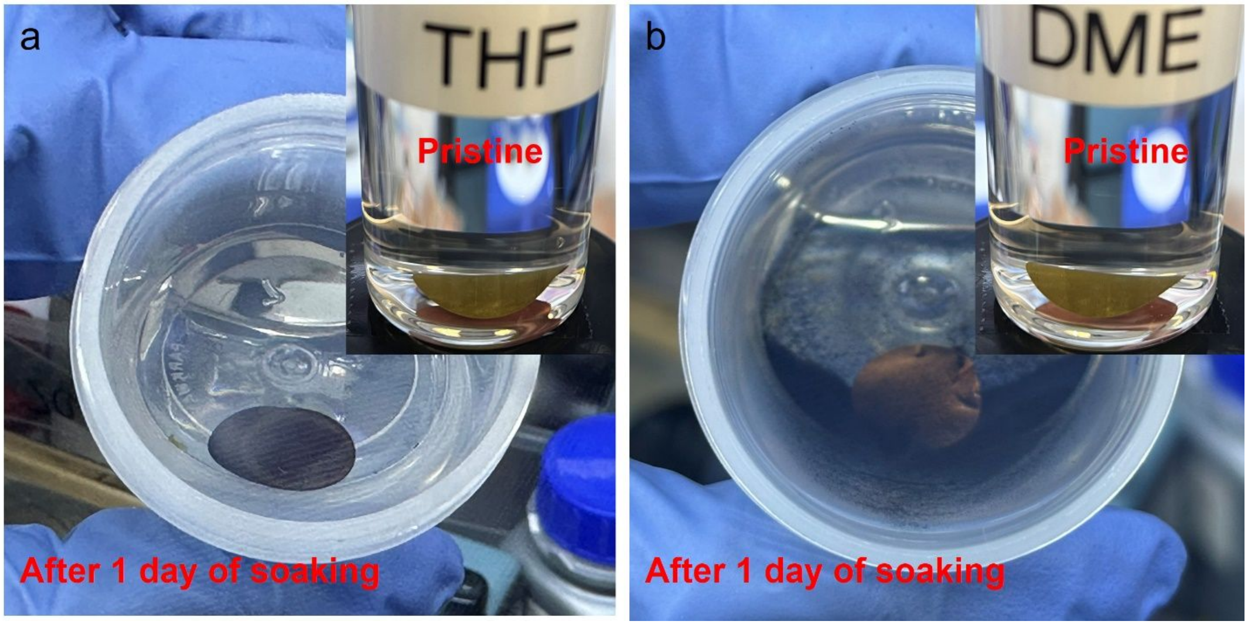

Fig. S14| LiC_6 electrode soaked in (**a**) THF and (**b**) DME solvents for 1 day. After 1 day of soaking, the color of both LiC_6 electrodes turned black. The electrode soaked in THF remained on the current collector even when shaken vigorously, while the electrode soaked in DME was peeled off and pulverized completely. This is because the volume expansion of t-DME-GIC is much larger than that of t-THF-GIC, which is confirmed with XRD data. LiC_6 was obtained from the G||Li cell lithiated to 0.005V at C/20 in the 1M $LiPF_6$-EC/EMC electrolyte.

For comparison, we also added another experiment to show the difference between DME and THF in transforming b-GIC to t-GIC. It is well known that DME has a strong chelating effect with Li-ion. As such, t-DME-GIC forms during the very first lithiation process. In this experiment, we demonstrated that the free DME solvent can undergo rapid chemical reactions with b-GIC. We made a LiC_6 electrode with a high mass loading (~7 mg/cm^2) and soaked it in DME and THF. After one day we took a photo and compared it with the original photo. The b-GIC to t-GIC reaction in THF is much less aggressive. Although the color of both graphite electrodes turned black, the graphite soaked in THF remained on the current collector even when shaken vigorously, while the LiC_6 electrode soaked in DME easily got peeled off and pulverized (Fig. S14).

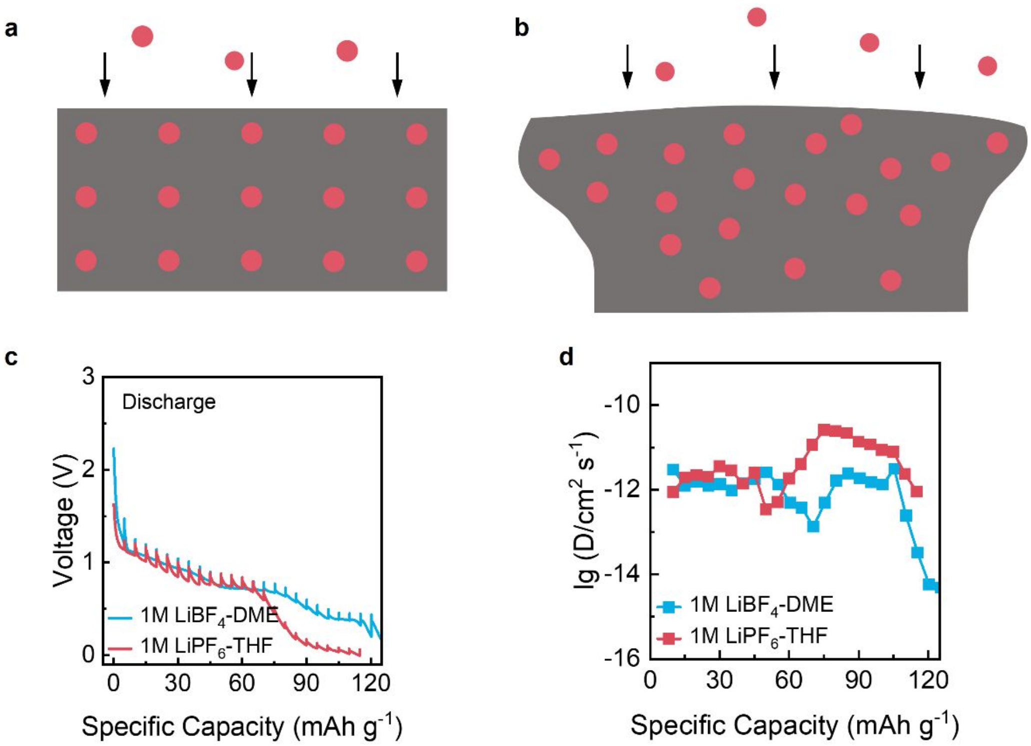

Fig. S15| a, Homogeneous distribution of intercalated species induces uniform volume expansion. **b**, The inhomogeneous distribution of intercalated species can induce mismatched strains in the host, which further induces inhomogeneous stresses. **c**, GITT curves of the G||Li cell cycled at different electrolytes. **d**, Corresponding calculated diffusion coefficients of the Li^+-THF and Li^+-DME in the graphite anode.

Moreover, the intercalation of molecules between graphite layers is accompanied by the expansion of the host lattice. This volume expansion induces stress if the deformation is constrained. Constraints may be geometric or may result from an inhomogeneous distribution of the intercalate species within graphite, which generates inhomogeneous strains within the particles. A homogenous distribution of intercalate species in an unconstrained host, as shown in Fig. S15a, would generate a uniform, stress-free expansion of the host. However, an inhomogeneous distribution of the intercalate species, as shown in Fig. S15b, generates a strain mismatch between the species-rich outer region and the inner region with a lower number of intercalate species. This inhomogeneous deformation generates a large compressive stress in the outer highly distended region and a tensile stress in the core that has a lower deformation. The degree of inhomogeneity depends on the competition between the rate of charging and the rate of diffusion. The significantly lower diffusion coefficient for DME in graphite, when compared to THF (Fig. S15c,d), induces

large gradients of concentration and, therefore, generates much larger mismatch strains than THF. Additionally, it is observed that graphite experiences a much larger volume expansion due to co-intercalation with DME when compared to THF (Fig. S13, S14). THF on the other hand, induces smaller concentration gradients and lower mismatch strains owing to its relatively higher diffusion coefficient and lower volume expansion. As a consequence of these factors, graphite with DME co-intercalation experiences much larger stresses and has a higher tendency of mechanical degradation. The higher mechanical stability is a major contributing factor to the better cyclic performance of THF when compared to co-intercalation with DME.

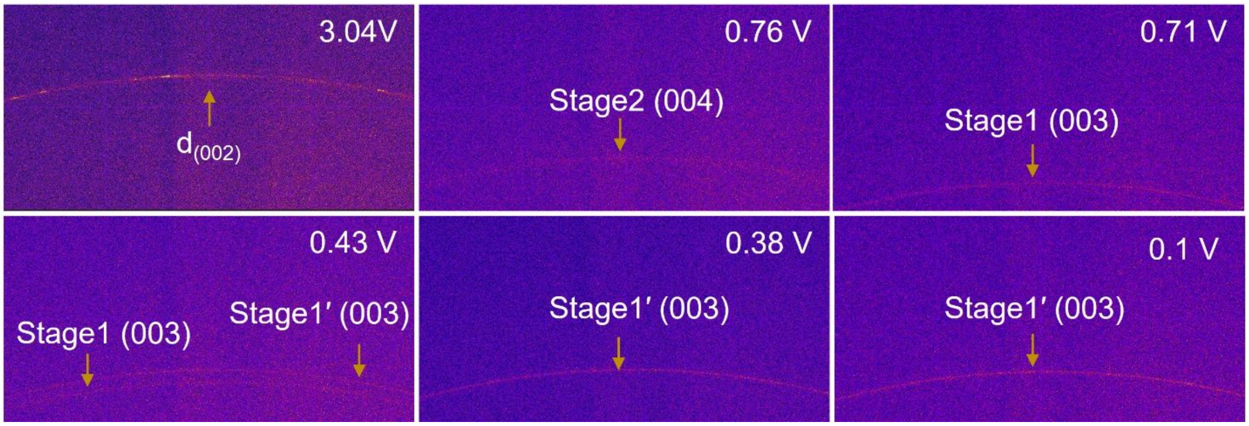

Fig. S16| CMCD of the G||Li cell cycled at 1C in the 1M $LiBF_4$-DME electrolyte (the 1st lithiation).

The CMCD measurement on some selected particles further shows that the t-DME-GIC formation takes place in the very first lithiation and all the studied graphite particles react concurrently. This causes a surge of volume and strain throughout the electrode. In contrast, the t-THF-GIC formation follows a non-concurrent fashion. Namely, some b-GICs (stage III) can form b-GICs (stage I, LiC_6), whereas some b-GICs transform to t-GICs by chemically reacting with THF (Figure 2c).

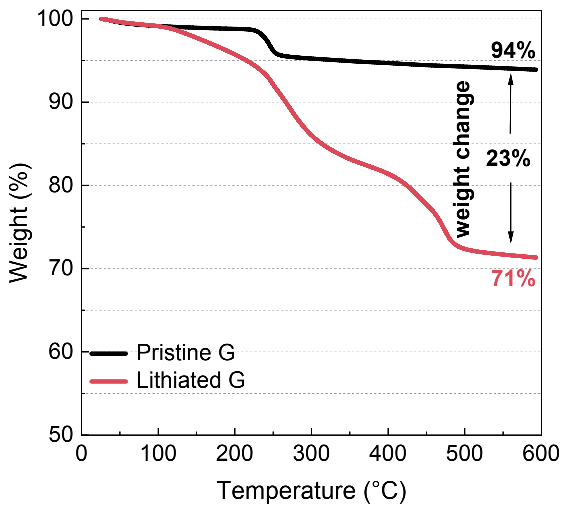

Fig. S17| TGA curves of the pristine and lithiated graphite electrodes (cycled in the 1M $LiPF_6$-THF electrolyte). Compared to pristine graphite, the weight changes of lithiated graphite are 23%, which is similar to the weight measurements (Figure 2d). The result supports that one THF molecule is co-intercalated with one Li^+. The weight loss in pristine graphite originates from sodium alginate.

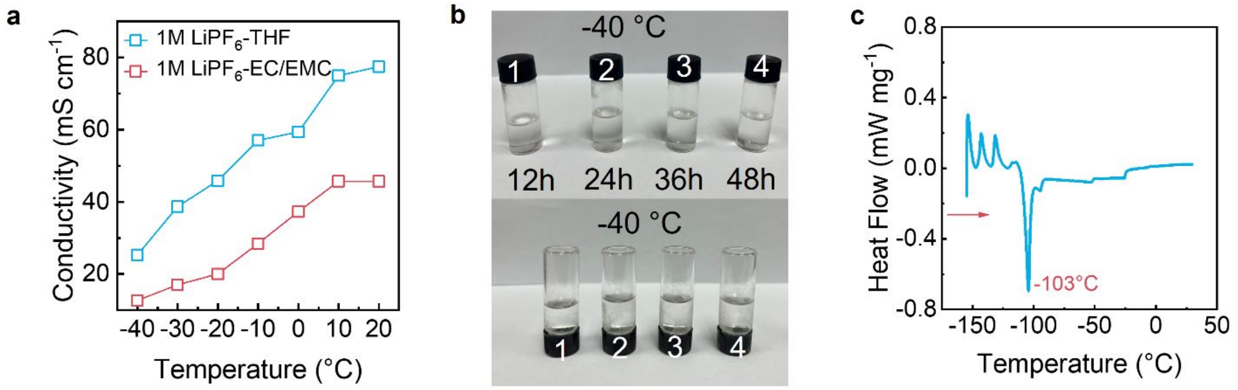

Fig. S18| a, The ionic conductivity of the electrolytes (1M $LiPF_6$-THF and 1M $LiPF_6$-EC/EMC) in the temperature range of -40 to 20 °C. **b**, Good fluidity and stability of the 1M $LiPF_6$-THF electrolyte at -40 °C. **c**, Differential Scanning Calorimetry (DSC) characterization of the 1M $LiPF_6$-THF electrolyte.

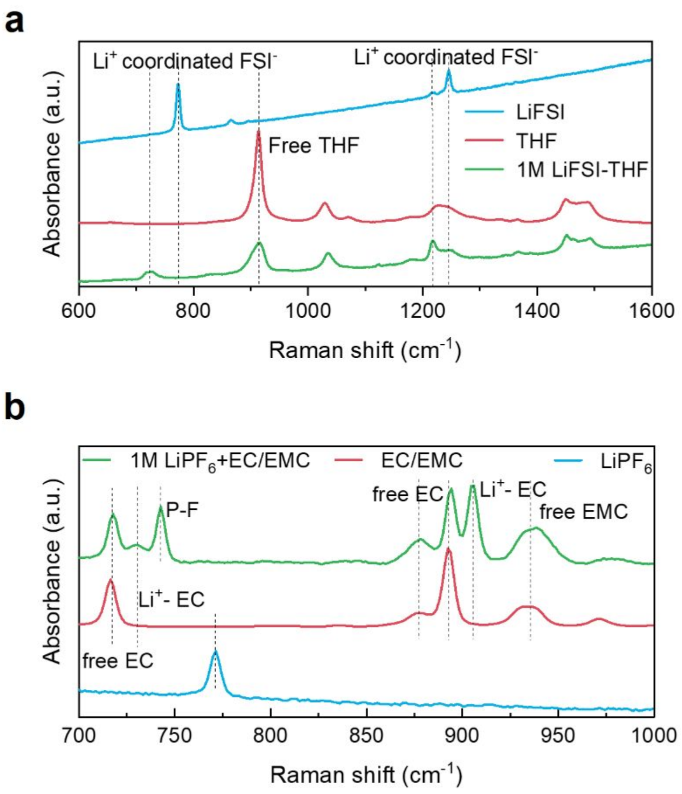

Fig. S19| Raman spectra of the: **a**, 1M LiFSI-THF electrolyte; **b**, 1M $LiPF_6$-EC/EMC electrolyte.

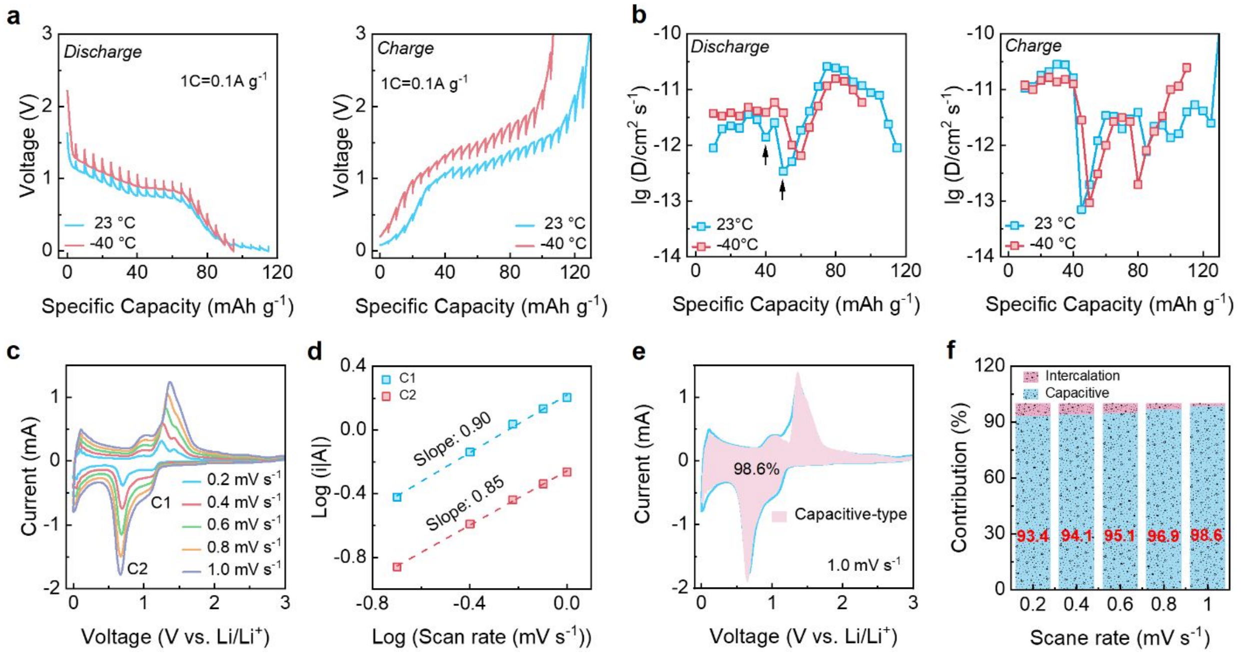

Fig. S20| Li$^+$-THF storage kinetics analysis. a, GITT curves of the G||Li cell cycled at different temperatures. **b,** Corresponding calculated diffusion coefficients of the Li$^+$-THF in the graphite anode. **c,** CV curves of the G||Li cell at different scan rates. **d,** b-value determination of the cathodic peak currents. **e**, CV curve at 1.0 mV s^{-1} with the fitted capacitive-type contribution. **f,** Intercalation and capacitive contributions of the G||Li cell at different scan rates.

To further investigate the fast kinetics of Li$^+$-THF storage, we conduct the galvanostatic intermittent titration technique (GITT) tests to calculate the diffusion properties at different temperatures (Fig. S14a). It is found that the $D_{Li^+\text{-THF}}$ of graphite is almost the same during cycling at 23 and -40 °C, which not only corroborates the excellent low-temperature performance but also verifies the fast Li$^+$-THF diffusion kinetics (Fig. S14b). To gain more fundamental insight into fast kinetics, the CV with various scan rates is analyzed (Fig. S14c). The cathodic peak currents are labeled as C1 and C2 in Fig. S14c. The relationship between peak current and scan rate can be calculated by $i = av^b$. Fig. S14d shows the plot of log(|i|) versus log (v), where the slope of the linear line corresponds to the b-value. A b-value of 0.5 generally indicates diffusion-controlled intercalation with slower diffusion kinetics, while a value of 1.0 indicates surface-limited capacitive reaction with faster kinetics[14]. As a result, both b values are closer to 1, which indicates the dominant surface-limited reactions (Fig. S14d). This is also indirectly verifying the GITT results, where the $D_{Li^+\text{-THF}}$ is low in the plateau regions (match with the C1 and C2) of the discharge

(Fig. 14a). However, this is only relatively low, where the capacitive behavior still dominates, indicating that the Li^+-THF diffusion kinetics is effectively accelerated in the graphite. The pseudocapacitive behavior of graphite electrodes could be attributed to the enlarged space of graphite during cycling. Furthermore, the contribution percentages of the two behaviors are estimated based on the method proposed by Dunn et al.[15], namely the following equation $i=k_1v+k_2v^{1/2}$. The results show that the capacitive reaction occupies 93.4, 94.1, 95.1, 96.9, and 98.6% at scan rates of 0.2, 0.4, 0.6, 0.8, and 1.0 mV s^{-1}, respectively (Fig. S14e and S14f). It is clearly illustrated that the Li^+-THF storage process in graphite is almost dominated by surface-limited capacitive reaction, further verifying fast diffusion kinetics.

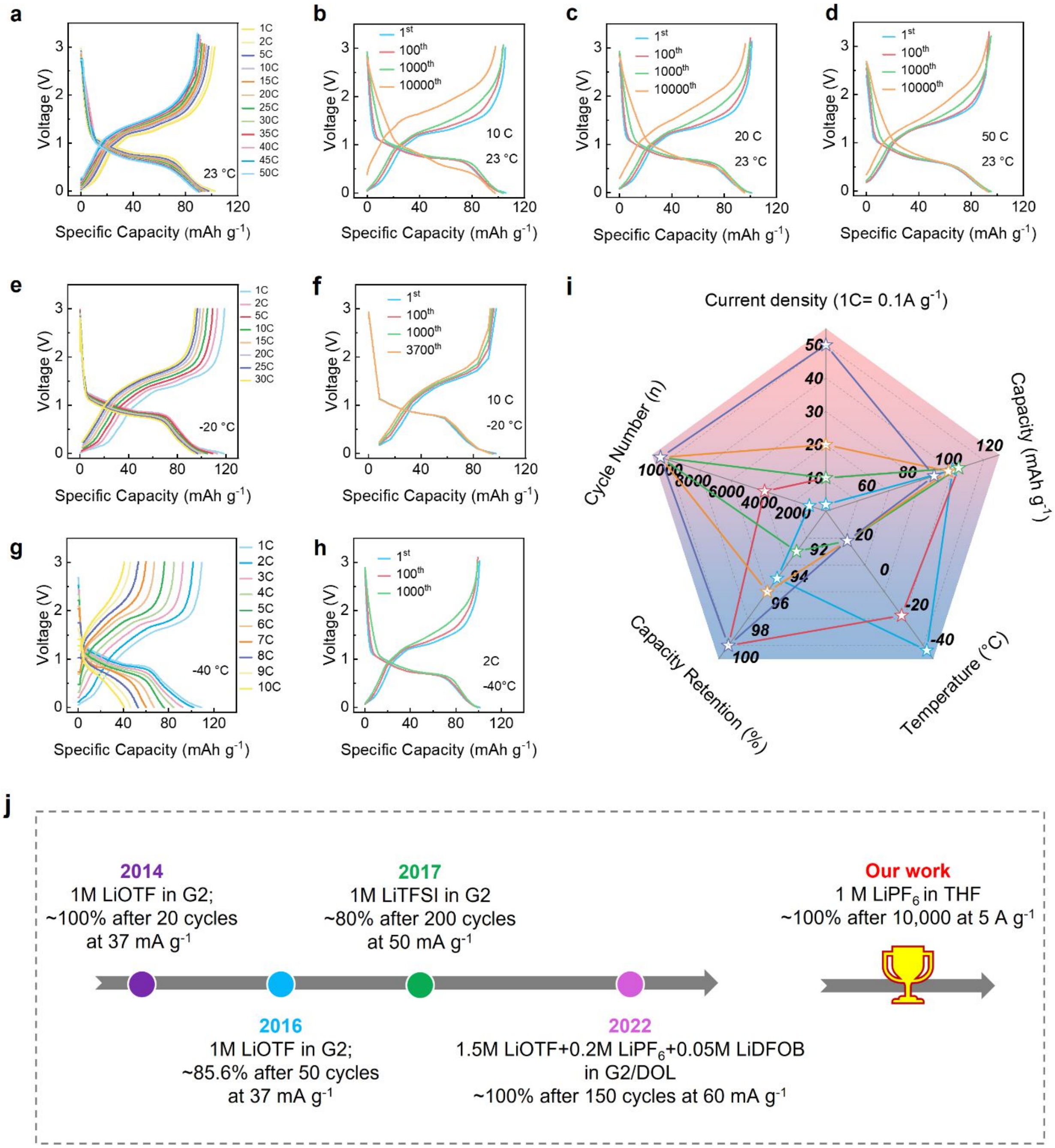

Fig. S21| Voltage profiles of the G||Li cell cycled at different C rates and temperatures: **a**, at the current range of 1 to 50 C under 23 °C. **b**, at 10 C under 23 °C. **c**, at 20 C under 23 °C. **d**, at 50 C under 23°C. **e**, at the current range of 1 to 30 C under -20 °C. **f**, at 10 C under -20 °C. **g**, at the current range of 1 to 10 C under -40 °C. **h**, at 2 C under -40 °C. **i**, The summary electrochemical data of the G||Li cell in the 1M $LiPF_6$-THF electrolyte at different currents and temperatures. **j,**

Comparison with the most successful co-intercalation electrolyte to date (more details are shown in Table S1)[16–19].

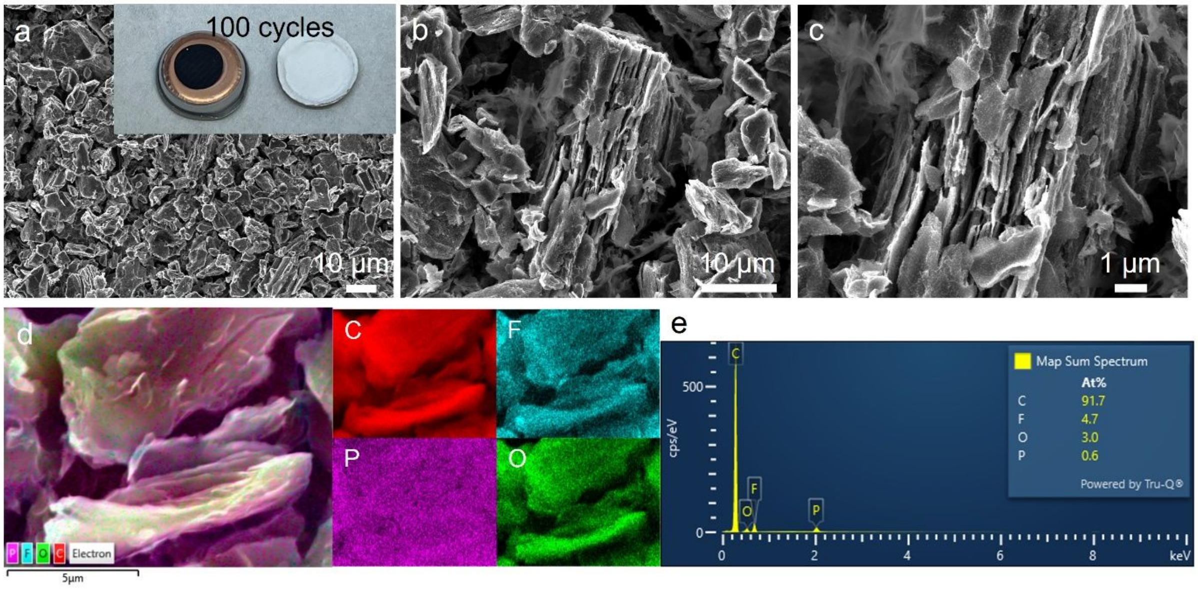

Fig. S22| a-c, SEM images of graphite anode cycled in the 1M $LiPF_6$-THF electrolyte at 10 C for 100 cycles. **d**, EDS mapping of C, F, P, O, and all elements. **e**, Atomic percent of each element. (Insert photograph is the disassembled cell after 100 cycles.)

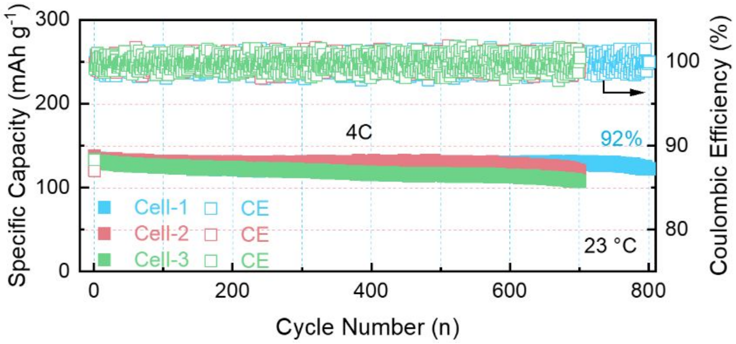

Fig. S23| Electrochemical performance of the G||NMC811 full cell in the 1M $LiPF_6$-THF electrolyte at 4 C under 23 °C, and the N/P ratio is 1.1.

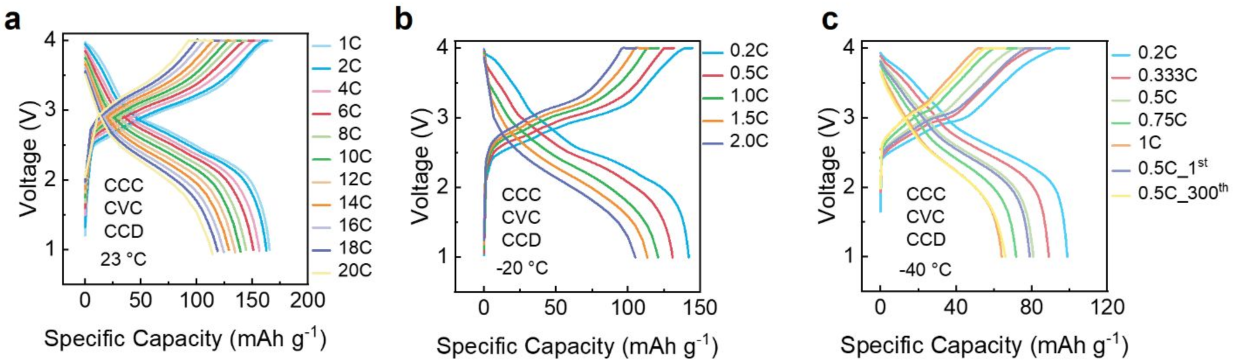

Fig. S24| Voltage profiles of the G||NMC811 full cell cycled at different C rates and temperatures: **a**, at the current range of 1 to 20 C under 23 °C. **b**, at the current range of 0.2 to 2.0 C under -20 °C. **c**, at the current range of 0.2 to 1 C under -40 °C.

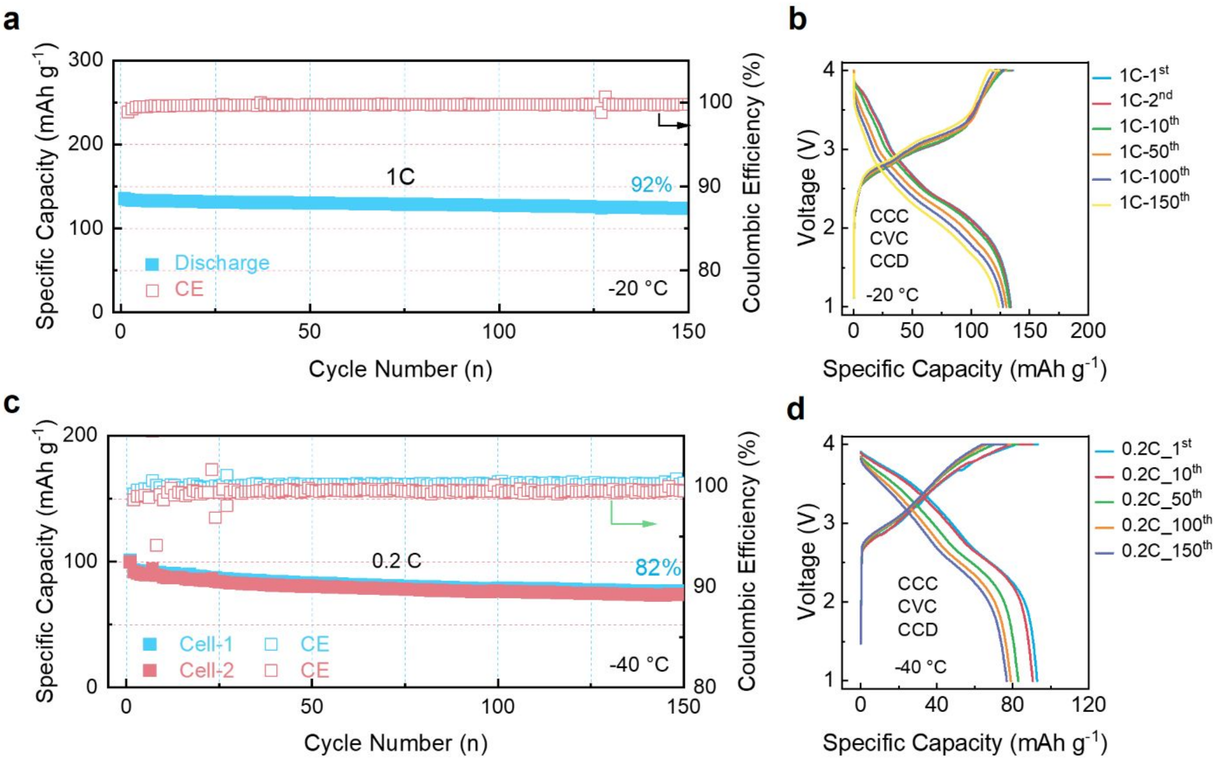

Fig. S25| Electrochemical performance of the G||NMC811 full cell in the 1M $LiPF_6$-THF electrolyte at low temperatures, and the N/P ratio is 1.1. **a-b**, Long-term cycling performance of the and corresponding voltage profiles of the G||NMC811 cell cycled at 1 C under -20 °C. **c-d,** Long-term cycling performance and corresponding voltage profiles of the G||NMC811 cell cycled at 0.2 C under -40 °C.

Table S1| Summary of the electrochemical performance of the solvent co-intercalation in graphite-based LIBs.

Cell configuration	Operating temperature	Electrolyte	Reversible capacity	Capacity retention	Ref./year
G‖Li[a]	23 °C	1 M $LiPF_6$-THF	~105 mAh g^{-1} at 10 C;<br>~100 mAh g^{-1} at 20 C;<br>~93 mAh g^{-1} at 50 C.	~93% after 10,000 cycles at 10 C;<br>~96% after 10,000 cycles at 20 C;<br>~100% after 10,000 cycles at 50 C.	**Our work (2023)**
	-20 °C		~101 mAh g^{-1} at 10 C.	~100% after 3,700 cycles at 10 C.	
	-40 °C		~102 mAh g^{-1} at 2 C	~95% after 1,000 cycles at 2 C.	
G‖NMC811[b]	23 °C		~133 mAh g^{-1} at 4 C;[c]<br>~146 mAh g^{-1} at 4 C.[d]	~92% after 800 cycles at 4 C;[c]<br>~97% after 400 cycles at 4 C[d].	
	-20 °C		~134 mAh g^{-1} at 1 C.[d]	~92% after 150 cycles at 1 C.[d]	
	-40 °C		~101 mAh g^{-1} at 0.2 C;[d]<br>~80 mAhg^{-1} at 0.5 C.[d]	~83% after 150 cycles at 0.2 C; [d]<br>~84% after 300 cycles at 0.5 C.[d]	
G‖Li[e]	25 °C	1.5 M LiOTF+0.2 M $LiPF_6$+0.05 M LiDFOB in DEGDME/DOL (1:1 vol)	~120 mAh g^{-1} at 0.5 C;	150 cycles at 0.5 C.	s19 (2022)
	-20 °C		~111 mAh g^{-1} at 0.5 C;	150 cycles at 0.5 C.	
	-40 °C		~93 mAh g^{-1} at 0.5 C;	150 cycles at 0.5 C.	
	-60 °C		~67 mAh g^{-1} at 0.5 C;	150 cycles at 0.5 C.	
G‖NMC[f]	25 °C		~106 mAh g^{-1} at 0.1 C. [d]	50 cycles at 0.1 C.	
	-20 °C		~116 mAh g^{-1} at 0.1 C. [d]	50 cycles at 0.1 C.	
	-40 °C		~108 mAh g^{-1} at 0.1 C; [d]<br>~80 mAh g^{-1} at 0.3 C. [d]	~78.5% after 100 cycles at 0.1 C;<br>~84.5% after 100 cycles at 0.3 C.	
	-60 °C		~62 mAh g^{-1} at 0.1 C. [d]	50 cycles at 0.1 C.	
G‖Li	RT	1 M LiTFSI-G2	~118 mAh g^{-1} at 50 mA g^{-1};<br>~100 mAh g^{-1} at 1 A g^{-1}.	/	*s18 (2017)*
G‖LFP	RT		~120 mAh g^{-1} at 50 mA g^{-1}	~80% after 200 cycles at 50 mA g^{-1}.	
G‖Li	RT	1 M LiOTF-G2	~100 mAh g^{-1} at 37 mA g^{-1}	~85.6% after 50 cycles at 37 mA g^{-1}.	*s17 (2016)*
G‖Li	RT	1 M LiOTF-G2	~100 mAh g^{-1} at 37 mA g-$^{-1}$	~100% after 20 cycles at 37 mA g^{-1}.	s16 (2014)

a: For the G||Li cell, the 1C=0.1 A g^{-1}. b: For the G||NMC811 cell, the 1C=0.2 A g^{-1}. c: Only a constant current charging (CCC) is performed for the G||NMC811 cell. d: A constant voltage charging (CVC) procedure is added to the CCC step. e: For G||Li cell, the 1C=0.12 A g^{-1}. f: For the G||NMC cell, the 1C=0.192 A g^{-1}. RT: room temperature.

Table S2| Comparison of the G||NMC811 full cell configurations with recently reported Na-/K-based full cell.

Cell configuration	[d]Pre-cycled G	Mass ratio (anode: cathode)	Voltage	Reversible capacity	[c]Energy and power densities	Ref
[a]G||NMC811	**No**	**1.4:1**	**2.7 V**	**~167 mAh g^{-1} at 0.2 A g^{-1}, ~114 mAh g^{-1} at 4.0 A g^{-1}**	**188 Wh kg^{-1} at 227 W kg^{-1}, 105 Wh kg^{-1} at 4180 W kg^{-1}**	***This work***
[a]G||$Na_{1.5}VPO_{4.8}F_{0.7}$	Pre-sodiated	1.5:1	3.1 V	~120 mAh g^{-1} at 0.05 A g^{-1}, ~96 mAh g^{-1} at 4.0 A g^{-1}	149 Wh kg^{-1} at 74.4 W kg^{-1}, 112 Wh kg^{-1} at 3863 W kg^{-1}	6 (2019)
[b]G||$Na_3V_2(PO_4)_2O_2F$	Pre-sodiated	2:1	3.2 V	~116 mAh g^{-1} at 0.05 A g^{-1}, ~66 mAh g^{-1} at 5.0 A g^{-1}	123.7 Wh kg^{-1} at 49.5 W kg^{-1}, 61.6 Wh kg^{-1} at 2464 W kg^{-1}	29 (2021)
[a]G||$Na_{0.7}CoO_2$	Pre-sodiated	3:1	2.2 V	~80 mAh g^{-1} at 0.175 A g^{-1}, ~40 mAh g^{-1} at 1.75 A g^{-1}	44 Wh kg^{-1} at 44 W kg^{-1}, 22 Wh kg^{-1} at 220 W kg^{-1}	30 (2016)
[b]G||$Na_3V_2(PO_4)_3$	n.d.	1:1.6	2.2 V	~90 mAh g^{-1} at 0.1 A g^{-1}, ~80 mAh g^{-1} at 2.0 A g^{-1}	76 Wh kg^{-1} at 76 W kg^{-1}, 67.7 Wh kg^{-1} at 1353.8 W kg^{-1}	31 (2015)
[b]G||$Na_{1.5}VPO_{4.8}F_{0.7}$	Pre-sodiated	1:1.5	2.9 V	~103 mAh g^{-1} at 0.1 A g^{-1}, ~40 mAh g^{-1} at 1.0 A g^{-1}	119 Wh kg^{-1} at 119 W kg^{-1}, 33.6 Wh kg^{-1} at 336 W kg^{-1}	37 (2015)
[a]MCMB||$Na_3V_2(PO_4)_3$	No	1:1	2.4 V	~51 mAh g^{-1} at 0.05 A g^{-1}, ~20 mAh g^{-1} at 15.0 A g^{-1}	64 Wh kg^{-1} at 75 W kg^{-1}, 44 Wh kg^{-1} at 5300 W kg^{-1}	32 (2022)
[a]G||$K_{1.94}Mn[Fe(CN)_6]_{0.994}\cdot 0.08H_2O$	Pre-potassiated	0.6:1	3.58 V	~145 mAh g^{-1} at 0.015 A g^{-1}, ~66.5 mAh g^{-1} at 0.4 A g^{-1}	331.5 Wh kg^{-1} at 33.1 W kg^{-1}, 145.4 Wh kg^{-1} at 387.9 W kg^{-1}	33 (2021)
[a]G||$K_{0.61}Fe[Fe(CN)_6]_{0.92}\cdot 0.32H_2O$	Pre-potassiated	1:1	2.9 V	~80 mAh g^{-1} at 0.05 A g^{-1}	116 Wh kg^{-1} at 58 W kg^{-1}	34 (2018)
[a]G||δ-$K_{0.51}V_2O_5$	Pre-potassiated	1:1	2.53 V	~94 mAh g^{-1} at 0.1 A g^{-1}, ~60 mAh g^{-1} at 2.0 A g^{-1}	119 Wh kg^{-1} at 119 W kg^{-1}, 75 Wh kg^{-1} at 1500 W kg^{-1}	*35 (2019)*
[b]G||$TiSe_2$ (Dual ion battery)	n.d.	3:1	2.5 V	~81.8 mAh g^{-1} at 0.1 A g^{-1}, ~45 mAh g^{-1} at 0.5 A g^{-1}	50.6 Wh kg^{-1} at 50.6 W kg^{-1}, 28 Wh kg^{-1} at 140 W kg^{-1}	36 (2021)

a: Capacity is calculated based on the mass of the cathode. b: Capacity is calculated based on the mass of the anode. c: Energy and power densities are calculated based on the mass of the cathode and anode. d: To decrease the irreversible capacity of the graphite, the graphite anode is pre-sodiated/pre-potassiated in some cases. However, this results in the presence of partial Na/K metal in the reaction, that is a hybrid full cell (Na ion/Na metal full cell, or K ion/K metal full cell), resulting in a high average voltage. References are cited in the main text.

Table S3. A short summary of the low-temperature performance of graphite-based LIBs[20–28].

Cell configuration	Operating temperature	Electrolyte	Reversible capacity	Capacity retention	Ref./year
G||NMC811	-40 °C	LiFSI/EMC/TTE (2:3.3:3.3 by mol)	~61.7 mAh g^{-1}	1 cycle at 0.1 C	*s20 (2022)*
G||NMC811	-20 °C	1 M $LiPF_6$-MP/FEC (9:1 vol%)	~95 mAh g^{-1}	100 cycles at 0.2 C	s21 (2021)
G||NMC523	-20 °C	1 M $LiPF_6$-EC/EMC/PC (4:7:1 wt%)+1 wt% $LiPO_2F_2$	~91.7mAh g^{-1}	~91% after 100 cycles at 0.5 C	s22 (2016)
G||NMC523	-20 °C	1 M $LiPF_6$-EC/EMC (1:2 wt%)+0.25 wt% LiDFBOP	~435 mAh	~93% after 50 cycles at 0.5 C	s23 (2018)
G||NMC523	-10 °C	1 M $LiPF_6$-EC/EMC (1:2 wt%)+0.5 wt% DMS	/	~98% after 50 cycles at 0.2 C	s24 (2019)
G||NMC622	-30 °C	1.3 M $LiPF_6$-BN/EC (3:1 vol%)+5 wt% FEC	~65 mAh g^{-1}	5 cycle at 0.1 C	s25 (2022)
G||NMC622	-20 °C	1 M $LiBF_4$-MA/FEC (9:1 vol%)	~96 mAh g^{-1}	after 50 cycles at 0.2 C	s26 (2022)
G||NMC811	-20 °C	0.5 M LiDFOB+0.5M $LiBF_4$+0.05 M $LiPF_6$+0.2 M LiDFP-MA/DFEC (9:1 vol%)	~167 mAh g^{-1}	~99% after 100 cycles at 0.2 C	s27 (2023)
G||NMC523	-20 °C	1 M LiFSI+50 mM NaFSI-DiFEC/MTFC/HFME (1:2:2 vol%)	~119 mAh g^{-1}	~92% after 100 cycles at 0.2 C	*s28 (2023)*
	-40 °C		~109 mAh g^{-1}	60 cycles at 0.05 C	
G||NMC811	-30 °C	1 M LiTFSI-MDFA/MDFSA/TTE (4:1:5 vol%)	~160 mAh g^{-1}	~82.8% after 345 cycles at 0.2 C	27 (2023)

References

(s1) Thompson, A. P.; Aktulga, H. M.; Berger, R.; Bolintineanu, D. S.; Brown, W. M.; Crozier, P. S.; in 't Veld, P. J.; Kohlmeyer, A.; Moore, S. G.; Nguyen, T. D.; Shan, R.; Stevens, M. J.; Tranchida, J.; Trott, C.; Plimpton, S. J. LAMMPS - a Flexible Simulation Tool for Particle-Based Materials Modeling at the Atomic, Meso, and Continuum Scales. *Comput. Phys. Commun.* **2022**, *271*, 108171.

(s2) McEldrew, M.; Goodwin, Z. A. H.; Kornyshev, A. A.; Bazant, M. Z. Theory of the Double Layer in Water-in-Salt Electrolytes. *J. Phys. Chem. Lett.* **2018**, *9* (19), 5840–5846.

(s3) Jorgensen, W. L.; Maxwell, D. S.; Tirado-Rives, J. Development and Testing of the OPLS All-Atom Force Field on Conformational Energetics and Properties of Organic Liquids. *J. Am. Chem. Soc.* **1996**, *118* (45), 11225–11236.

(s4) Becke, A. D.; Becke; D., A. Density-Functional Thermochemistry. III. The Role of Exact Exchange. *J. Chem. Phys.* **1993**, *98* (7), 5648–5652.

(s5) McLean, A. D.; Chandler, G. S. Contracted Gaussian Basis Sets for Molecular Calculations. I. Second Row Atoms, Z=11–18. *J. Chem. Phys.* **1980**, *72* (10), 5639–5648.

(s6) Frisch, M. J.; Pople, J. A.; Binkley, J. S. Self-consistent Molecular Orbital Methods 25. Supplementary Functions for Gaussian Basis Sets. *J. Chem. Phys.* **1984**, *80* (7), 3265–3269.

(s7) Singh, U. C.; Kollman, P. A. An Approach to Computing Electrostatic Charges for Molecules. *J. Comput. Chem.* **1984**, *5* (2), 129–145.

(s8) Besler, B. H.; Merz, K. M.; Kollman, P. A. Atomic Charges Derived from Semiempirical Methods. *J. Comput. Chem.* **1990**, *11* (4), 431–439.

(s9) Canongia Lopes, J. N.; Pádua, A. A. H. CL&P: A Generic and Systematic Force Field for Ionic Liquids Modeling. *Theor. Chem. Acc.* **2012**, *131* (3), 1–11.

(s10) Jewett, A. I.; Stelter, D.; Lambert, J.; Saladi, S. M.; Roscioni, O. M.; Ricci, M.; Autin, L.; Maritan, M.; Bashusqeh, S. M.; Keyes, T.; Dame, R. T.; Shea, J. E.; Jensen, G. J.; Goodsell, D. S. Moltemplate: A Tool for Coarse-Grained Modeling of Complex Biological Matter and Soft Condensed Matter Physics. *J. Mol. Biol.* **2021**, *433* (11), 166841.

(s11) Martínez, L.; Andrade, R.; Birgin, E. A Package for Building Initial Configurations for Molecular Dynamics Simulations. *J. Comput. Chem*. **2009**, *30* (13), 2157–2164.

(s12) Humphrey, W.; Dalke, A.; Schulten, K. VMD: Visual Molecular Dynamics. *J. Mol. Graph.* **1996**, *14* (1), 33–38.

(s13) Slater, M. D.; Kim, D.; Lee, E.; Johnson, C. S. Sodium-Ion Batteries. *Adv. Funct. Mater.* **2013**, *23* (8), 947–958.

(s14) Tao, L.; Sittisomwong, P.; Ma, B.; Hu, A.; Xia, D.; Hwang, S.; Huang, H.; Bai, P.; Lin, F. Tailoring Solid-Electrolyte Interphase and Solvation Structure for Subzero Temperature, Fast-Charging, and Long-Cycle-Life Sodium-Ion Batteries. *Energy Storage Mater.* **2023**, *55*, 826–835.

(s15) Wang, J.; Polleux, J.; Lim, J.; Dunn, B. Pseudocapacitive Contributions to Electrochemical Energy Storage in TiO_2 (Anatase) Nanoparticles. *J. Phys. Chem. C* **2007**, *111* (40), 14925–14931.

(s16) Jache, B.; Binder, J. O.; Abe, T.; Adelhelm, P. A Comparative Study on the Impact of Different Glymes and Their Derivatives as Electrolyte Solvents for Graphite Co-Intercalation Electrodes in Lithium-Ion and Sodium-Ion Batteries. *Phys. Chem. Chem. Phys.* **2016**, *18*, 14299.

(s17) Jache, B.; Adelhelm, P. Use of Graphite as a Highly Reversible Electrode with Superior Cycle Life for Sodium-Ion Batteries by Making Use of Co-Intercalation Phenomena. *Angew. Chem. Inter. Ed.* **2014**, *53* (38), 10169–10173.

(s18) Kim, H.; Lim, K.; Yoon, G.; Park, J. H.; Ku, K.; Lim, H. D.; Sung, Y. E.; Kang, K. Exploiting Lithium–Ether Co-Intercalation in Graphite for High-Power Lithium-Ion Batteries. *Adv. Energy Mater.* **2017**, *7* (19), 1700418.

(s19) Yang, Y.; Chen, Y.; Tan, L.; Zhang, J.; Li, N.; Ji, X.; Zhu, Y. Rechargeable $LiNi_{0.65}Co_{0.15}Mn_{0.2}O_2$||Graphite Batteries Operating at −60 °C. *Angew. Chem. Int. Ed.* **2022**, *61* (42), e202209619.

(s20) Nan, B.; Chen, L.; Rodrigo, N. D.; Borodin, O.; Piao, N.; Xia, J.; Pollard, T.; Hou, S.; Zhang, J.; Ji, X.; Xu, J.; Zhang, X.; Ma, L.; He, X.; Liu, S.; Wan, H.; Hu, E.; Zhang, W.; Xu, K.; Yang, X. Q.; Lucht, B.; Wang, C. Enhancing Li+ Transport in NMC811||Graphite Lithium-Ion Batteries at Low Temperatures by Using Low-Polarity-Solvent Electrolytes. *Angew. Chem. Int. Ed.* **2022**, *61* (35), e202205967.

(s21) Cho, Y. G.; Li, M.; Holoubek, J.; Li, W.; Yin, Y.; Meng, Y. S.; Chen, Z. Enabling the Low-Temperature Cycling of NMC||Graphite Pouch Cells with an Ester-Based Electrolyte. *ACS Energy Lett.* **2021**, *6* (5), 2016–2023.

(s22) Yang, B.; Zhang, H.; Yu, L.; Fan, W. Z.; Huang, D. Lithium Difluorophosphate as an Additive to Improve the Low Temperature Performance of $LiNi_{0.5}Co_{0.2}Mn_{0.3}O_2$/Graphite Cells. *Electrochim. Acta* **2016**, *221*, 107–114.

(s23) Liao, B.; Li, H.; Xu, M.; Xing, L.; Liao, Y.; Ren, X.; Fan, W.; Yu, L.; Xu, K.; Li, W.; Liao, B.; Li, H.; Xu, M.; Xing, L.; Liao, Y.; Li, W.; Ren, X.; Fan, W.; Yu, L.; Xu, K. Designing Low Impedance Interface Films Simultaneously on Anode and Cathode for High Energy Batteries. *Adv. Energy Mater.* **2018**, *8* (22), 1800802.

(s24) Guo, R.; Che, Y.; Lan, G.; Lan, J.; Li, J.; Xing, L.; Xu, K.; Fan, W.; Yu, L.; Li, W. Tailoring Low-Temperature Performance of a Lithium-Ion Battery via Rational Designing Interphase on an Anode. *ACS Appl. Mater. Interfaces* **2019**, 11(41), 38285-38293.

(s25) Yoo, D. J.; Liu, Q.; Cohen, O.; Kim, M.; Persson, K. A.; Zhang, Z. Understanding the Role of SEI Layer in Low-Temperature Performance of Lithium-Ion Batteries. *ACS Appl. Mater. Interfaces* **2022**, *14* (9), 11910–11918.

(s26) Rodrigo, N. D.; Jayawardana, C.; Rynearson, L.; Hu, E.; Yang, X.-Q.; Lucht, B. L. Use of Ethylene Carbonate Free Ester Solvent Systems with Alternative Lithium Salts for Improved Low-Temperature Performance in NCM622|| Graphite Li-Ion Batteries. *J. Electrochem. Soc.* **2022**, *169* (11), 110504.

(s27) Liang, J.-Y.; Zhang, Y.; Xin, S.; Tan, S.-J.; Meng, X.-H.; Wang, W.-P.; Shi, J.-L.; Wang, Z.-B.; Wang, F.; Wan, L.-J.; Guo, Y.-G. Mitigating Swelling of the Solid Electrolyte Interphase Using an Inorganic Anion Switch for Low-Temperature Lithium-Ion Batteries. *Angew. Chem. Int. Ed.* **2023**, *135* (16), e202300384.

(s28) Zheng, X.; Cao, Z.; Luo, W.; Weng, S.; Zhang, X.; Wang, D.; Zhu, Z.; Du, H.; Wang, X.; Qie, L.; Zheng, H.; Huang, Y.; Zheng, X.; Luo, W.; Wang, D.; Zhu, Z.; Du, H.; Cao, Z.; Zheng, H.; Weng, S.; Wang, X.; Zhang, X.; Qie, L.; Huang, Y. Solvation and Interfacial Engineering Enable −40 °C Operation of Graphite/NCM Batteries at Energy Density over 270 Wh Kg^{-1}. *Adv. Mater.* **2023**, *35* (10), 2210115.
